# T-cell Intracellular Antigen (TIA)-Proteins Deficiency in Murine Embryonic Fibroblasts Alters Cell Cycle Progression and Induces Autophagy

**DOI:** 10.1371/journal.pone.0075127

**Published:** 2013-09-24

**Authors:** Carmen Sánchez-Jiménez, José M. Izquierdo

**Affiliations:** Centro de Biología Molecular Severo Ochoa, Consejo Superior de Investigaciones Científicas, Universidad Autónoma de Madrid (CSIC/UAM), Madrid, Spain; Konkuk University, Korea

## Abstract

Mice lacking either T-cell intracellular antigen 1 (TIA1) or TIA1 related/like protein (TIAR/TIAL1) show high rates of embryonic lethality, suggesting a relevant role for these proteins during embryonic development. However, intrinsic molecular and cellular consequences of either TIA1 or TIAR deficiency remain poorly defined. By using genome-wide expression profiling approach, we demonstrate that either TIA1 or TIAR inactivation broadly alter normal development-associated signalling pathways in murine embryonic fibroblasts (MEF). Indeed, these analyses highlighted alterations of cytokine-cytokine and ECM-receptor interactions and Wnt, MAPK, TGF-beta dependent signalling pathways. Consistent with these results, TIA1 and TIAR knockout (KO) MEF show reduced rates of cell proliferation, cell cycle progression delay and increased cell size. Furthermore, TIA-proteins deficiency also caused metabolic deficiencies, increased ROS levels and DNA damage, promoting a gentle rise of cell death. Concomitantly, high rates of autophagy were detected in both TIA1 and TIAR KO MEF with induction of the formation of autophagosomes, as evidenced by the up-regulation of the LC3B protein, and autolysosomes, measured by colocalization of LC3B and LAMP1, as a survival mechanism attempt. Taken together, these observations support that TIA proteins orchestrate a transcriptome programme to activate specific developmental decisions. This program is likely to contribute to mouse physiology starting at early stages of the embryonic development. TIA1/TIAR might function as cell sensors to maintain homeostasis and promote adaptation/survival responses to developmental stress.

## Introduction

The T-cell intracellular antigen 1 (TIA1) and TIA1 related/like (TIAR/TIAL1) proteins were initially identified associated to nucleolysins and polyadenylate binding proteins localized to the granules of cytolytic lymphocytes and involved in apoptosis by DNA fragmentation [1], [2]. These proteins are RNA-binding proteins highly conserved in mammals with structural and functional homologs in other eukaryotic organisms, thus revealing the ancestral importance of these functional regulators across the evolution [3]–[5].

TIA1 and TIAR are multifunctional proteins that modulate many aspects of RNA metabolism -both in the nucleus and cytoplasm- at different regulatory levels of gene expression. For example, they modulate DNA-dependent transcription by interacting with DNA and RNA polymerase II [6]–[9], they control alternative splicing of pre-mRNA (around 10% of splicing events in human) by facilitating the selection of atypical 5′ spliced sites [10]–[13] and they also regulate stability and/or translation of eukaryotic mRNAs by binding to the 5′ and/or 3′ untranslatable regions [13]–[22].

TIA proteins are known to target genes with relevant biological implications in apoptosis, inflammation, cell responses to stress, viral infections and oncogenesis [1], [2], [18], [20]–[24]. Further, these proteins seem to have an important role during embryogenesis. For example, mice lacking either TIA1 or TIAR, as well as ectopically over-expressing TIAR, show higher rates of embryonic lethality [18], [25], [26].

Although the role of TIA proteins in key cellular processes involving inflammatory and the stress responses are well established, their roles on developmental and patho-physiological programs have not been elucidated yet. In this work, we approach the characterization of molecular and cellular phenoypes associated to the TIA1 or TIAR knocked-out murine embryonic fibroblast (MEF) cells. Our results point out that TIA proteins control cell cycle and proliferation and provide evidence suggesting that they function as cellular sensors controlling autophagy and cell death responses.

## Materials and Methods

### Cell cultures and reagents

Immortalized murine embryonic fibroblast wild type knock-out for either TIA1 or TIAR [18], [25] were maintained as described previously [27]. For protein labelling, MEF cells incubated with methionine-cysteine free DMEM supplemented with 5 µl Easy Tag™ EXPRESS [^35^S] Protein Labeling mix, [^35^S]-Met-Cys (11 mCi/ml, 37.0 Tbq/mmol; Perkin Elmer) for 30 min. To inhibit autophagy, MEFs were treated with 10 µM chloroquine (CQ) (Sigma) for 96 h. For hydrogen peroxide (H_2_O_2_) treatment, MEF cells were incubated with the indicated H_2_O_2_ concentrations in normal medium for 6 hours or 3 days.

### Preparation of cell extracts and western blot analysis

Whole-MEF cell extracts were performed and processed as described previously [27]. Immunoblots were carried out using the following antibodies: anti-TIA1 and anti-TIAR (Santa Cruz Biotechnology), anti-α-tubulin (Sigma), anti-U2AF65 (kindly provided by J. Valcárcel), anti-Cdc-2 and anti-Cdc2-P (Y15) (Cell Signaling), anti-Cyclin B1 (BD Pharmingen), anti-LC3B (Sigma), anti-p62 (Sigma) and anti-LAMP1 (DSHB).

### DNA purification, RNA isolation, semiquantitative and quantitative RT-PCR analysis

DNA purification was performed using DNeasy Blood and Tissues kit (Qiagen). Total RNA isolation, semiquantitative RT-PCR and quantitative PCR analysis were carried out as described previously [20], [27].

### Transcriptome analysis

RNA quality check, amplification, labelling, hybridization with Array SurePrint Mouse G3 8×60 (Agilent, G4852A) and initial data extraction were performed at the Genomic Service Facility at the Centro Nacional de Biotecnología (CNB-CSIC). Comparison of multiple cDNA array images (two independent experiments per biological condition tested) was carried out by using an average of all of the gene signals on the array (global normalization) to normalize the signal between arrays. Local background was corrected by normexp method with an offset of 50. Background corrected intensities were transformed to log scale (base 2) and normalized by loess for each array [28]. Finally, to have similar intensity distribution across all arrays, loess-normalized-intensity values were scaled by adjusting their quantiles [29]. After data processing each probe was tested for changes in expression over replicates using an empirical Bayes moderated t statistic [30]. To control the false discovery rate (FDR), *P* values were corrected using the method of Benjamini and Hochberg (1995) [31]. FIESTA viewer (http://bioinfogp.cnb.csic.es/tools/ FIESTA) was used to visualize all microarray results and to evaluate the numerical thresholds (−2>Fold change>2; FDR LIMMA<0.05) applied for selecting differentially expressed genes [32]. Microarray data discussed in this publication have been deposited in the NCBI Gene Expression Omnibus database (http://www.ncbi.nlm.nih.gov/ geo/info/linking.html) and are accessible through the GEO Series accession number GSE43077. The Gene Ontology (GO) and Kyoto Encyclopedia of Genes and Genomes (KEGG) database analyses were conducted using software programmes provided by GenCodis3 (http://genecodis.cnb. csic.es) [33], [34].

The validation of microarray analysis for TIA1/TIAR-regulated genes was carried out by quantitative PCR (QPCR) using SYBR green method. Reverse transcriptase (RT) reactions and real-time PCR (PCR) were performed according to manufacturer protocols at the Genomic PCR Core Facility at the Centro de Biología Molecular Severo Ochoa (CBMSO-CSIC). Analysis were performed in two independent samples by triplicate, including no-template and RT-minus controls. GAPDH expression was used as endogenous reference control. Relative mRNA expression was calculated using the comparative cycle threshold method.

### Cell and growth proliferation

For cell proliferation analysis, wild-type MEF and TIA1 or TIAR knocked-MEF were seeded (1.5 10^4^/well) in six-well plates and collected for counting at the indicated time points. Cell growth was quantified by measuring the conversion of methyl thiazolyl tetrazolium (MTT) (Sigma) into DMSO-soluble formazan by living cells, with absorbance measured at 570 nm using a spectrophotometer [23].

### Analysis of the cell-cycle in not-synchronized and synchronized MEFs

Cell-cycle analysis was carried out by flow cytometry after fixation in 70% Ethanol for 24 h and propidium iodide staining (BD Pharmingen). MEFs were synchronized by serum starvation for 48–96 h or by serum starvation for 24 h followed by incubation with 1 mM Hydroxyurea (Sigma) for 16–24 h or 200 ng/ml Nocodazole (Sigma) for 24–30 h.

### Fluorescence microscopy

MEF cells were grown for 24 h on coverslips, washed with phosphate-buffered saline (PBS), fixed in Formalin (Sigma) at room temperature for 10 min, washed with PBS, and processed. For immunofluorescence, the coverslips were incubated for 45 min at room temperature with the primary antibody diluted in PBS solution (Phalloidin (Sigma [1∶500]), anti-LAMP1 (DSHB [1∶50]) or LC3B (Sigma [1∶100]). The samples were then washed with PBS and incubated for 45 min with the corresponding secondary antibody (Invitrogen [dilution 1∶500]) plus To-Pro-3 (Invitrogen [1∶1000]). The samples were then washed in PBS and mounted with Mowiol (Calbiochem). Manders' colocalization coefficients (identified as M1 and M2) were calculated using the intensity correlation analysis plugin for ImageJ software. After background subtraction, threshold was determined by Huang's algorithm [35]. Immunodetection of oxidative damage within nuclear DNA was carried using a mouse monoclonal antibody identified as anti-8-oxo-dG (Clone 2E2), which specifically binds to 8-hydroxy-2′-deoxyguanosine, according to the manufacturer's instructions (Trevigen). The mitochondrial populations were illustrated by using 500 nM Mito Tracker Green FM (Invitrogen) for 30 min before fixation. The microscopic observations were carried out by using a confocal microscope.

### Electron microscopy

MEFs were grown for 24 h and fixed *in situ* with 4% paraformaldehyde and 0.1% glutaraldehyde in Sörensen phosphate buffer (pH 7.4) for 90 min at room temperature. Fixed cells were washed three times in phosphate Na/K buffer (pH 7.4), removed from the dishes and transferred to eppendorf tubes. After centrifugation, cells were processed for embedding in Epoxy, TAAB 812 Resin (TAAB Laboratories, Berkshire, England) according to standard procedures. Postfixation of cells was done with a mixture 1% osmium tetroxide and 0.8% potassium ferrocyanide in bidistilled water for 1 h at 4°C. After three washes with bidistilled water samples were incubated with 0.15% tanic acid in buffer for 1 min at room temperature. After several washes with buffer and bidistilled water, cells were incubated with 2% uranyl acetate for 1 h at room temperature, washed again and dehydrated in increasing concentrations of ethanol (50, 70, 90, 95 and 100%) for 5 min each at 4°C. Infiltration of the resin was accomplished in increasing concentrations of Epon-ethanol (1∶2.1∶1.2∶1 and 100% Epon) at room temperature for 1 day. Polymerization of infiltrated samples was done at 60°C during 2 days. Ultrathin sections of the samples were stained with saturated uranyl acetate and lead citrate by standard procedures and examined at 80 Kv in a Jeol JEM-1010 (Tokyo) electron microscope.

### SA-β-galactosidase staining

The expression of senescence-associated β-galactosidase (SA-β-gal) in MEF cells was determined by using a SA-β-gal staining kit (Senescence cells histochemical staining kit (Sigma) according to manufacturer's instructions.

### FACS analysis

Cell death rates were quantified by using Annexin V: PE apoptosis detection kit (BD Pharmingen) according to manufacturer's protocol. Mitochondrial parameters were determined using the following probes: 100 nM Mito Tracker Green FM (Invitrogen) for 25 min, 100 nM tetramethylrhodamine methylester (TMRM) (Sigma) for 15 min, and 5 µM 2′, 7′-dichlorodihydrofluorescein diacetate (H2DCFDA) (Invitrogen) for 30 min.

### Estimation of the mitochondrial DNA (mtDNA) copy number

We performed quantitative PCR by using SYBR green and total DNA template. We used the following primers (Sigma): mtCO1 primers, 5-CCCAATCTCTACCAGCATC-3 and 5-GGCTCATAGTATAGCTGGAG-3; mtND1 primers, 5-AATCGCCATAGCCTTCCTAACAT-3 and 5-GGCGTCTGCAAATGGTTGTAA-3; nTNF primers, 5-TCCCTCTCATCAGTTCTATGGCCCA-3 and 5-CAGCAAGCATCTATGCACTTAGACCCC-3; nH19 primers: 5-GTACCCACCTGTCGTCC-3 and 5-GTCCACGAGACCAATGACTG-3 [36]. We normalized the amount of mtDNA to the amount of the nuclear DNA (nDNA).

### Statistical analysis

Represented values are shown as means + standard error of the mean (SEM). Differences were tested for significance by means of the Student's *t*-test. A probability level *P*<0.05 was considered significant.

## Results

### High-throughput gene expression profiling of either TIA1 or TIAR-knocked murine embryonic fibroblasts

**Figure 1:**
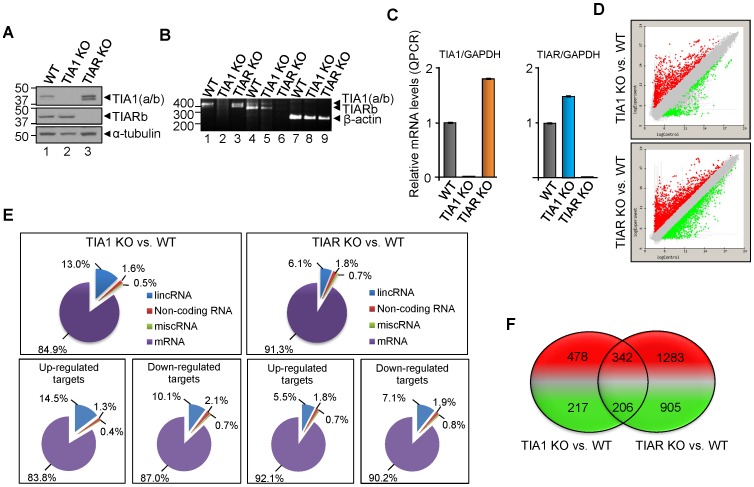
Characterization of the transcriptomes associated to the knockout (KO) of TIA1 or TIAR in murine embryonic fibroblast (MEF) cells. (A) Western blot analysis of wild-type (WT), TIA1 KO and TIAR KO MEF cell extracts (10 µg of total protein). The blot was probed with antibodies against TIA1, TIAR and α-tubulin proteins, as indicated. Molecular weight markers and the identities of protein bands are shown. (B) Cytoplasmic mRNAs from above MEF cells were analyzed by semiquantitative RT-PCR. Positions of size markers and the predicted alternatively spliced products are indicated. (C) Quantification of relative levels of TIA1, TIAR and GAPDH mRNAs in the above MEF cells by real time RT-PCR. The represented values were normalized and expressed relative to GAPDH. (D) MA plot representation of the distribution of up- (spots in red) and down-regulated (spots in green) RNAs (−2>fold-change<2; FDR<0.05) in either TIA1 or TIAR KO MEF versus WT MEF by using Array SurePrint Mouse G3 8×60 (Agilent, G4852A). (E) Graphic representations of the distribution of up- and down-regulated target genes in TIA1 or TIAR KO MEF versus WT MEF. In all cases, percentages shown reflect the portion of total genes that are associated with the RNA categories corresponding to long non-coding RNAs (lncRNA), non-coding RNAs, miscellaneous RNAs (miscRNA) and messenger RNAs (mRNA). (F) Venn diagram depicting the numbers of genes that were up- (red) and down-regulated (green) as well as shared between both categories of TIA1 or TIAR knocked out MEF.

To get new insights into the role of TIA1 and TIAR proteins during embryonic development we decided to test the transcriptome of murine embryonic fibroblasts (MEF) lacking either TIA1 (TIA1 KO MEF) or TIAR (TIAR KO MEF) proteins [18], [25]. The absence of TIA1 and TIAR mRNAs and proteins in the TIA1 and TIAR KO MEFs is illustrated by western blotting (Fig. 1A) and semiquantitative and quantitative RT-PCR analyses (Fig. 1B and C), confirming previous results [18], [25], [27]. Global RNA expression patterns in either TIA1 KO MEF or TIAR KO MEF compared to wild-type (WT) MEF were made using a mouse genome-wide microarray (Agilent SurePrint Mouse G3 8×60, version G4852A) from two different biological samples for each experimental condition tested (Fig. 1D-F). To identify cohorts of mouse RNAs regulated by TIA proteins, the gene expression array dataset was analyzed using an appropriate statistical test analysis, which was made using a linear model (as implemented in the limma R/Biocounductor package) to compare RNA expression patterns. In these analysis, only RNAs showing at least a −2>fold change>2 (FDR<0.05) in expression compared to the WT MEF were considered. As shown in Figure 1D, TIA1- and TIAR-deficiency resulted in a marked alteration in gene expression compared to the normal expression patterns of wild-type MEFs (FIESTA viewer software in http:// bioinfogp. cnb.csic.es/tools/FIESTA). To address the functional relevance of differentially expressed RNAs in TIA1 and TIAR KO MEF cells versus wild-type MEF cells, the long intergenic non-coding RNA (lincRNA), non-coding RNA, miscellaneous RNA (miscRNA) and protein encoding-RNA (mRNA) are shown (Fig. 1E and Fig. S1 and S2). While similar gene expression patterns were observed when TIA and TIAR KO MEF samples were compared, it is noteworthy the increase in up-regulated lincRNAs that is associated to TIA1-deficiency. A total of 1243 (of which 820 and 423 were up- and down-regulated, respectively) and 2736 (of which 1625 and 1111 were up- and down-regulated, respectively) RNAs were differentially expressed (FDRlimma<0.05) in TIA1 and TIAR KO MEF cells, respectively (Fig. 1F and Fig. S1 and S2). Of them, 342 up- and 206 down-regulated RNAs were common in both TIA1- and TIAR-deficient MEFs (Fig. 1F and Fig. S3). Additionally, we have found 13 down-regulated RNAs in TIA1 KO MEF cells which were up-regulated in TIAR KO MEF cells, whereas 40 up-regulated RNAs in TIA1 KO MEF cells were down-regulated in TIAR KO MEF cells (Fig. S3). Collectively, these results indicate that TIA1 and TIAR regulate both specific and overlapping aspects of the mouse embryonic transcriptome, suggesting that their functional roles can be either redundant, additive and/or independent, in agreement with previous findings [20].

**Figure 2:**
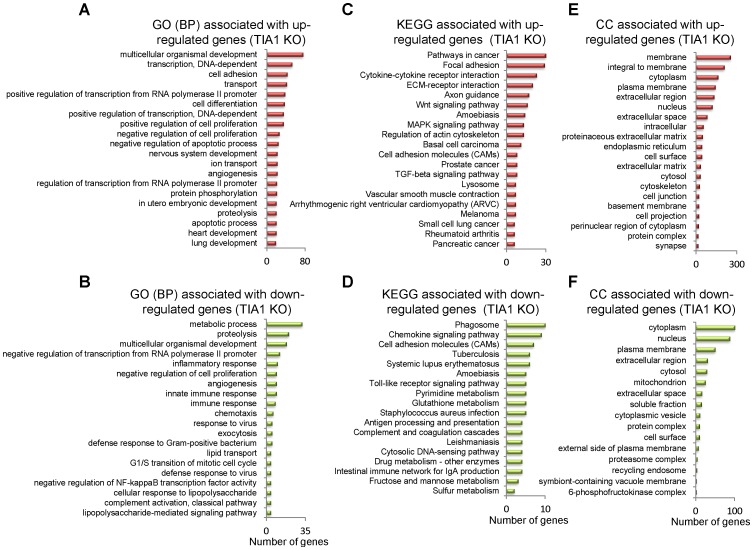
Top-twenty categories of biological processes, pathways and cellular components associated to the TIA1 KO MEF. (A and B) Histograms of the distribution of up- (A) and down-regulated (B) genes using the Gene Ontology (GO) biological category (*P*<0.05). (C and D) Histograms of the distribution of up- (C) and down-regulated (D) genes using the Kyoto Encyclopedia of Genes and Genomes (KEGG) pathway database (*P*<0.05). (E and F) Histograms of the distribution of up- (E) and down-regulated (F) genes using the cellular component (CC) database (*P*<0.05).

As a first attempt to understand the functional relevance of differentially expressed up- and down-regulated genes in TIA and TIAR KO MEF cells, Gene Ontology (GO) and Kyoto Encyclopedia of Genes and Genomes (KEGG) database analysis were performed. GO analysis was able to identify the main categories of biological processes of differentially expressed RNAs controlled by TIA proteins (*P*<0.05) (Fig. 2A and B and Fig. S4). GO categories related to multicellular organismal development, positive/negative regulation of transcription, cell adhesion, transport, cell differentiation, negative/positive regulation of cell proliferation, negative regulation of apoptotic process, nervous system development, angiogenesis and protein phosphorylation were among the enriched categories in up-regulated genes by the absence of TIA1 (Fig. 2A and Fig. S4). In contrast, GO categories associated with metabolic process, proteolysis, multicellular organismal development, negative regulation from RNA polymerase II promoter, inflammatory response, negative regulation of cell proliferation, angiogenesis, innate immune response, chemotaxis and response to virus were especially prevalent among down-regulated genes (Fig. 2B and Fig. S4).

KEGG database analysis integrating individual components into unified pathways was used to identify the enrichment of specific pathways in functionally regulated gene groups (Fig. 2C and D and Fig. S4). The results show that several KEGG pathways were significantly enriched (*P*<0.05) in up-regulated genes, including pathways involved in cancer, focal adhesion, cytokine-cytokine receptor interaction, EMC-receptor interaction, axon guidance, Wnt signalling pathway, amoebiasis, MAPK signalling pathway and regulation of actin cytoskeleton (Fig. 2C and Fig. S4). The top ten of KEGG pathways significantly enriched (*P*<0.05) in down-regulated genes include phagosome function, chemokine signalling pathways, cell adhesion molecules, tuberculosis, systemic lupus erythematosus, amoebiasis, Toll-like receptor signalling pathway, pyrimidine metabolism, gluthatione metabolism and *Staphylococcus aureus* infection (Fig. 2D and Fig. S4). From a cellular viewpoint, the cell components associated to the up- and down-regulated genes in TIA KO MEF were located on cellular compartments connected to membrane, cytoplasm and nucleus (Fig. 2E and F and Fig. S4). Taken together, these results suggest the existence of severe alterations in programs controlling multicellular organism development and in signal transduction pathways, followed by pathways regulating specific cellular, inflammatory, immune and metabolic responses that might be counteracting/contributing to the embryonic phenotype associated to the TIA1 KO MEF.

**Figure 3:**
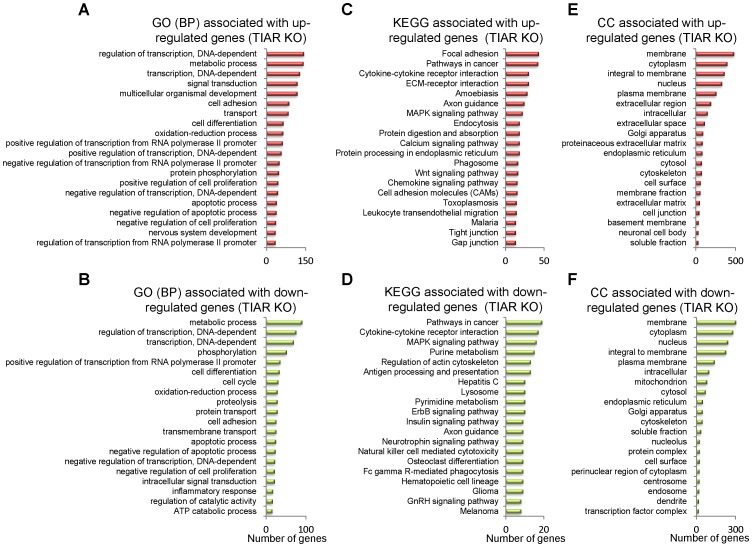
Top-twenty categories of biological processes, pathways and cellular components associated to the TIAR KO MEF cells. (A and B) Histograms of the distribution of up- (A) and down-regulated (B) genes using the Gene Ontology (GO) biological category (*P*<0.05). (C and D) Histograms of the distribution of up- (C) and down-regulated (D) genes using the Kyoto Encyclopedia of Genes and Genomes (KEGG) pathway database (*P*<0.05). (E and F) Histograms of the distribution of up- (E) and down-regulated (F) genes using the cellular component (CC) database (*P*<0.05).

GO biological process categories and KEGG pathway analyses of differentially expressed genes were also performed in TIAR KO MEF (Fig. 3 and Fig. S5). GO categories (*P*<0.05) associated to up-regulated genes were related with transcriptional regulation, metabolic processes, signal transduction, multicellular organism development, cell adhesion, transport, cell differentiation, oxidative pathways, protein phosphorylation and regulation of cell proliferation (Fig. 3A and Fig. S5). In contrast, GO categories (*P*<0.05) of down-regulated genes associated with the absence of TIAR in MEF were linked to genes involved in metabolic processes, transcriptional regulation, phosphorylation, cell differentiation, cell cycle, oxidative pathways, proteolysis, protein transport, cell adhesion and apoptosis (Fig. 3B and Fig. S5). KEGG data (*P*<0.05) for up-regulated genes in TIAR KO MEF cells were associated with genes related to focal adhesion, tumorigenesis, cytokine-cytokine receptor interaction, ECM-receptor interaction, amoebiasis, axon guidance, MAPK signalling pathway, endocytosis, protein digestion and absorption, and calcium signalling pathway (Fig. 3C and Fig. S5). KEGG categories for down-regulated genes in TIAR-depleted MEF cells were related to pathways in cancer, cytokine-cytokine receptor interaction, MAPK signalling pathway, purine metabolism, regulation of actin cytoskeleton, antigen processing and presentation, hepatitis C, lysosome biology, pyrimidine metabolism and ErbB signalling pathway (Fig. 3D and Fig. S5). From a cellular viewpoint, the cell components associated to the up- and down-regulated genes in TIAR KO MEF cells were located on cellular compartments connected to membrane, cytoplasm and nucleus (Fig. 3E and F and Fig. S5). Thus, these results suggest that both in TIAR and TIA1 regulate both specific and overlapping aspects of the mouse embryonic transcriptome related to the developmental program of multicellular organisms and signal transduction pathways that contribute to the embryonic phenotypes associated to the MEF cells.

To know whether knocked-down expression of TIA proteins is associated with the regulation of common gene clusters, we tested by GO and KEGG database analysis all genes that were identified to be up or down regulated by both TIA1 and TIAR deficiency (Fig. S3). The results indicate that TIA1 and TIAR shared targets that are related with multicellular organism development, transcriptional regulation, cell adhesion, signal transduction, metabolism processes, proteolysis inflammation and angiogenesis.

### Validation of microarray-predicted changes in gene expression

The effects on steady-state RNA levels detected by the array analysis were validated using quantitative PCR assays for 11 different RNAs. Figure S6 shows validation of predicted up-regulated (FBN2, MEST, SFRP1, SFRP2 and XIST) and down-regulated (ARG2, EREG and HMGA2) RNAs in TIA1 and TIAR KO MEFs, respectively, as well as the sequences of the primers used in the amplification. As expected, the results confirmed the array data (Fig. S6). GAPDH expression level was used as normalizer in this study of validation.

### TIA1 or TIAR-knocked MEF cells show reduced rates of cell proliferation and morphological transformation

**Figure 4:**
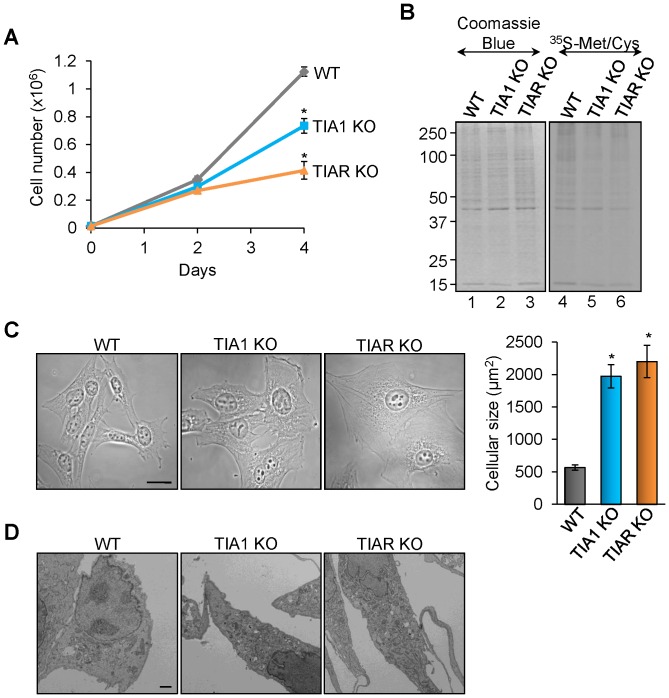
MEF cells knocked for TIA proteins show cell proliferation defects and rise cellular size and complexity. (A) Wild-type (WT), TIA1 KO and TIAR KO MEF cells were grown and the number was counted on the days indicated. Each time point represents the means + standard error of the mean (SEM; n = 6; *P<0.001). (B) Nascent translation of total proteins was determined by incubation of wild-type (WT), TIA1 KO and TIAR KO MEF in the presence of ^35^S-methionine/cystein mix. The expression patterns of total proteins (15 µg) and newly translated proteins from above MEF cells were visualized by staining with Coomassie Blue reagent and by 10% SDS-PAGE and autoradiography, respectively. (C) Representative phase contrast photographs of WT, TIA1 KO and TIAR KO MEFs are shown. Scale bars represent 20 µm. The cellular size of WT, TIA1 KO and TIAR KO MEFs was quantified using ImageJ software and represented as means + SEM (n = 15; *P<0.001). (D) Representative transmission electron micrographs of WT, TIA1 KO and TIAR KO MEFs are exposed. Scale bars represent 3 µm.

GO and KEGG analysis suggest that the absence of TIA1 and TIAR proteins has a repercussion on cell proliferation rates in MEF (Fig. 2 and 3 and Fig. S3–S5). Thus, we examined the proliferation potential of MEF with knocked down expression of either TIA1 or TIAR. As shown in Figure 4A, the absence of TIA1 or TIAR expression in MEF resulted in decreased cell proliferation compared to control MEF. Indeed, total cell numbers as well as measurement of de novo synthesized proteins by [^35^S]-methionine and -cystein incorporation support an individual role for each of these TIA proteins in the negative control of MEF proliferation (Fig. 4A and B). Given that the rates of TIA1 and TIAR KO MEF proliferation were reduced, we analyzed the morphology and size of these cells by optical and electronic microscopy. The results illustrate that TIA1 and TIAR KO MEF have a significant morphological alteration compared to normal MEFs, showing a larger cellular size (3-4 fold) and a more complex cytoplasm than WT MEF (Fig. 4C and D and Fig. S7).

### TIA1 or TIAR-knocked MEF cells show anomalies affecting mitochondrial biology

**Figure 5:**
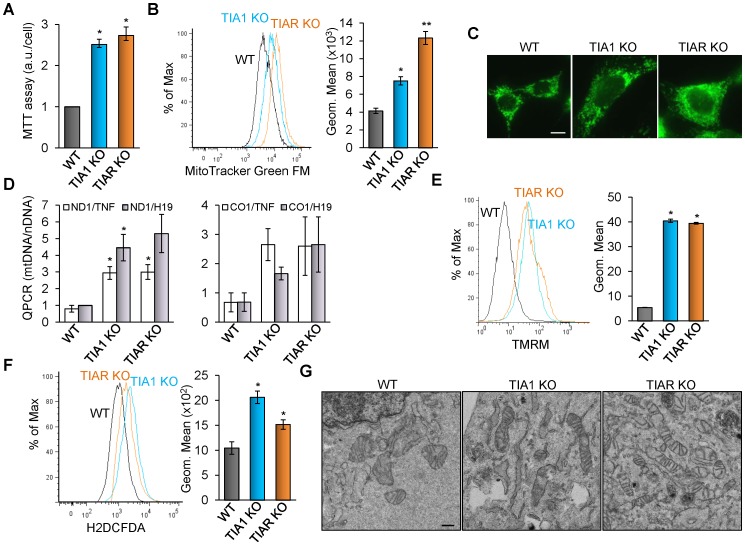
MEF cells lacking TIA proteins show mitochondrial alterations. (A) Wild-type (WT), TIA1 KO and TIAR KO MEFs were grown for 4 days and monitored by methyl thiazolyl tetrazolium (MTT) assays. The represented values were normalized and expressed relative to WT, whose value was fixed arbitrarily to 1, and are means + SEM (n = 4; *P<0.001). (B) Wild-type (WT), TIA1 KO and TIAR KO MEFs were stained with Mito Tracker Green FM and monitored by FACS analysis. The represented values are means + SEM (n = 3; *P<0.01; **P<0.001). (C) Mitochondrial populations of wild-type (WT), TIA1 KO and TIAR KO MEF were stained with Mito Tracker Green FM and visualized by confocal microscopy. Scale bars represent 10 µm. (D) Mitochondrial DNA copy number was estimated by quantitative PCR (QPCR) using mitochondrial NADH subunit 1 (ND1) and cytochrome c oxidase subunit 1 (CO1) and nuclear tumor necrosis factor (TNF) and H19 as markers for the copy numbers of mitochondrial DNA (mtDNA) and nuclear DNA (nDNA), respectively. The represented values were normalized and expressed relative to WT, whose value was fixed arbitrarily to 1, and are means + SEM (n = 4; *P<0.05). (E) Estimation of the mitochondrial membrane potential of wild-type (WT), TIA1 KO and TIAR KO MEFs by staining with TMRM molecular probe and monitoring by FACS analysis. The represented values are means + SEM (n = 3; *P<0.001). (F) Estimation of reactive oxygen species (ROS) of wild-type (WT), TIA1 KO and TIAR KO MEFs by staining with H2DCFDA molecular probe and monitoring by FACS analysis. The represented values are means + SEM (n = 3; *P<0.01). (G) Transmission electron micrographs of cytoplasmic sections from wild-type (WT), TIA1 KO and TIAR KO MEFs illustrating the mitochondrial morphology and integrity. Scale bars represent 400 nm.

To expand previous results and to determine the cellular processes underlying the biological features associated to TIA1 and TIAR deficiency, we measured the metabolic activity of MEFs by methyl thiazolyl tetrazolium (MTT) assay. The results suggest that TIA1 and TIAR KO MEFs have higher metabolic rates (2-3-fold) than WT MEFs associated with cell reductase activity (Fig. 5A). As the MTT assay reflects indirectly the cell growth capacity of cells as function of the mitochondria metabolism, we decided to quantify several mitochondrial parameters such as mitochondrial mass/distribution, activity and morphology of WT, TIA1 KO, and TIAR KO MEFs. The relative mitochondrial mass as well as mitochondria cell distribution were determined by Mito Tracker Green TM staining of WT, TIA1 KO and TIAR KO MEFs either by flow cytometry or by fluorescence microscopy, respectively. The results indicated that TIA1 and TIAR KO MEF had more abundance (2-3-fold) of mitochondria than WT MEF (Fig. 5B and C). In addition, we estimated the relative mitochondria number as the ratio between the amount of mitochondrial DNA (mtDNA) and nuclear DNA (nDNA), measured by quantitative PCR using specific primers against two mitochondrial DNA-encoded genes (ND1 and CO1) and nuclear DNA-encoded genes (TNF and H19) [36]. The results suggest that TIA1 and TIAR KO MEFs had 2-3-fold more mitochondria that WT MEFs (Fig. 4D). Taken together, these observations are consistent with the existence of a more abundant mitochondrial population in TIA1 and TIAR KO MEF which is consistent with the larger cellular size found in these murine embryonic cells.

Next, we determined in vivo the mitochondrial membrane potential (ΔΨ) using the fluorescence probe TMRM and flow cytometry [37]. The results show that mitochondrial populations of TIA1 and TIAR KO MEFs had 6-7-fold larger mitochondria membrane potential compared to mitochondria in WT MEF cells (Fig. 5E). This result suggests the existence of functional anomalies in the mitochondria of TIA1 and TIAR deficient MEFs, since considering that these MEFs have 2-3 fold more mitochondria than control MEFs, the observed increase of mitochondrial membrane potential is twice higher than that expected. To test whether this increase in ΔΨ could be the consequence of alterations in mitochondrial metabolism, for example an anomalous production of reactive oxygen species (ROS), we quantified the levels of ROS using in vivo staining with the fluorescence probe H2DCFDA followed by flow cytometry. The results indicated that ROS levels were increased twice in TIA1 and TIAR KO MEFs compared to WT MEF (Fig. 5F). These observations suggest a malfunction of mitochondrial metabolism as a result of either TIA1 or TIAR deficiency. To illustrate this functional abnormality we visualized the mitochondrial morphology and integrity by electron microscopy. The results show the existence of mitochondria with atypical enlarged morphology as well as many broken mitochondria in TIA1 and TIAR KO MEF cells (Fig. 5G and Fig. S8). Collectively, this collection of observations points out that TIA1 and TIAR KO MEF versus WT MEF contain several abnormal mitochondrial parameters such as increased mitochondrial mass and number, altered morphology and integrity, increased membrane potential and overproduction of ROS, all of them indicative of an altered mitochondrial metabolism.

### Extensive oxidative damage of nuclear DNA in TIA1 and TIAR KO MEF cells

**Figure 6:**
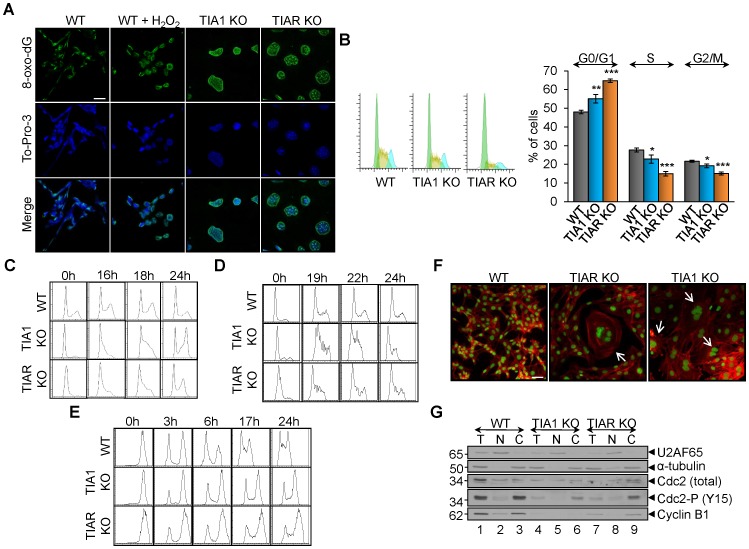
MEF cells lacking TIA proteins show oxidized nuclear DNA and G2/M phase delay during cell-cycle progression. (A) Visualization of genomic DNA oxidized in wild-type (WT), TIA1 KO and TIAR KO MEFs by indirect immunofluorescence using an anti-8-oxo-dG antibody. WT MEFs were treated with 100 µM H_2_O_2_ for 20 min to promote genomic DNA oxidation as a positive control. Scale bars represent 15 µm. (B) Analysis of cell-cycle phases by flow cytometry after propidium iodide staining. The data are means + SEM (n = 10; *P<0.05; **P<0.01; ***P<0.001). (C) MEF cells knocked for TIA proteins showed delayed entry into G1/S. The above MEF cells were synchronized at G0/G1 by serum deprivation for 48–96 h. Samples were taken at 0, 16, 18 and 24 h after release, and the DNA content was measured by propidium iodide staining and FACS analysis. (D) MEF cells knocked for TIA proteins showed delayed entry into S. The above MEF cells were synchronized at G1/S by Hydroxyurea blockage for 16–24 h and then released. Samples were taken at 0, 19, 22 and 24 h after release, and the DNA content was measured by propidium iodide staining and FACS analysis. (E) MEF cells knocked for TIA proteins showed delayed entry into G0/G1. The above MEF cells were synchronized at G2/M by Nocodazole blockade for 24–30 h and then released. Samples were taken at 0, 3, 6, 17 and 24 h after release, and the DNA content was measured by propidium iodide staining and FACS analysis. (F) Examples of MEF cells stained with To-Pro-3 (illustrated in green) and phalloidin-TRITC showing impairment of cytokinesis events in TIA1 and TIAR KO MEF. Scale bars represent 20 µm. (G) Analysis of the G2/M DNA damage checkpoint. Immunoblot of total (T), nuclear (N) and cytoplasmic (C) fractions (8 µg) from wild-type (WT), TIA1 KO and TIAR KO MEFs. The blot was probed with antibodies against U2AF65, α-tubulin, Cdc2 (total), Cdc2-P (Y15) and cyclin B1 proteins, as indicated. Molecular weight markers and the identities of protein bands are shown.

To evaluate the potential deleterious effect of the excess of ROS production found in TIA1 and TIAR KO MEFs on the integrity of nuclear DNA, we have carried out an experimental approach of immunofluorescence microscopy using anti-8-oxo-dG antibody. The 8-hydroxy-2′-deoxyguanosine (8-oxo-dG) is a modified nucleoside by-product commonly used to detect DNA damage caused by oxidative radicals [38]. As shown in Figure 6A, labelling of the nuclei of TIA1 and TIAR KO MEFs with the anti-8-oxo-dG was readily observed, compared with WT MEFs. As a control, we induced oxidative DNA damage with H_2_O_2_ of WT MEF (Fig. 6A). These observations suggest that the nuclear DNA of MEFs lacking either TIA1 or TIAR are massively damaged by the cellular ROS and/or the inactivation of repairing pathways.

### Cell-cycle altered progression in TIA1 and TIAR KO MEF cells

Given that the knockout of either TIA1 or TIAR results in defects in cell proliferation, cell size and causes damage of nuclear DNA, we next examined whether TIA1/TIAR-deficiency had deleterious effects on cell-cycle progression that could account for the cell proliferation defects observed for TIA1 and TIAR knocking. Our results showed that not-synchronized TIA1 and TIAR-deficient MEF cells already evidenced a modest impact on the cell-cycle progression by increasing G0/G1 and diminishing S and G2/M transitions, compared to WT MEFs, suggesting a defect of the progression and through phases and/or the transition from G1, S and/or G2/M (Fig. 6B). To further characterize this phenotype, we analyzed the cell-cycle progression of cells released from a cell cycle blockage at G0/G1 after 48–96 h of culture in the absence of fetal calf serum. As shown in Figure 6C, TIA1 and TIAR knocked-down MEF cells showed a significant increase in cells at late S phase (see 18 h after release), suggesting a slower transition through S phase. In addition, we analyzed the cell cycle progression of cells released from a single blockage at G1/S with Hydroxyurea (16–24 h) (Fig. 6D) or at G2/M with Nocodazole (24–30 h) (Fig. 6E). In both cases, we observed a delay in S and G1 entries in both TIA1 and TIAR deficient MEF cells, indicative of a defect in the transition from G1 to S and G2/M to G1, respectively (Fig. 6D and E). Representative images of multinucleated cells with impaired cytokinesis are shown in Figure 6F as well as the quantification and cellular localization of Cdc-2 and cyclin B1 protein markers illustrating the steady-state of G2/M checkpoint (Fig. 6G). TIA1 and TIAR KO MEFs showed the lower expression levels of these cell cycle regulators. Taken together, these data suggest that either TIA1 or TIAR knockout in MEFs have a mitosis defect leading to an accumulation of cells in G2/M.

### Immortalized TIA1 and TIAR knocked MEF cells show low senescence

**Figure 7:**
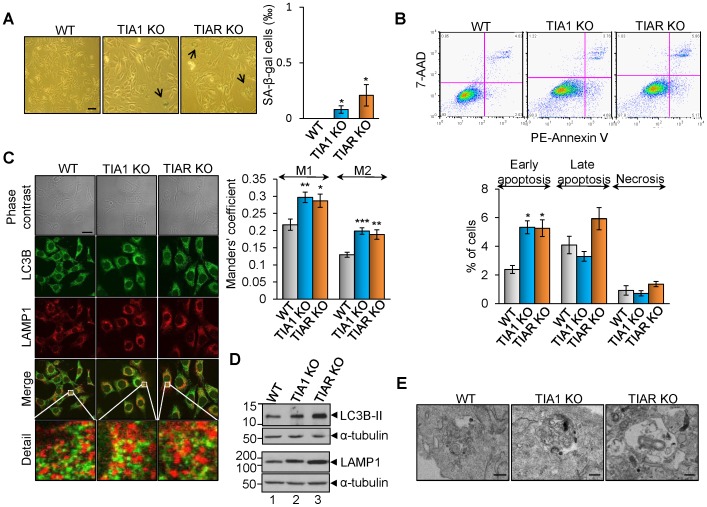
TIA1 and TIAR KO MEFs show high rates of autophagy. (A) Senescence analysis with SA-β-gal staining. Wild-type (WT), TIA1 KO1 and TIAR KO MEF cells were subjected to SA-β-gal staining and quantified of positively stained cells. The represented values are means + SEM (n = 12 fields; *P<0.05). Scale bars represent 40 µm. (B) Analysis of cell death rates. Rates of apoptosis (early and late) and necrosis were quantified by using 7-AAD staining and PE Annexin V apoptosis detection kit followed by flow cytometry analysis. The graph shows the percentage of cells counted that were in each stage of cell death. The represented values are means + SEM (n = 9; *P<0.001). (C) Visualization of autophagosomes and lysosomes in wild-type (WT), TIA1 KO and TIAR KO MEFs cells by indirect immunofluorescence using anti-LC3B and anti-LAMP1 antibodies. The extended detail illustrates the degree of cellular co-localization between both markers as evidence of autolysosomes. The overlapping degree between signals was quantified using Manders' M1 and M2 colocalization coefficients. We can therefore interpret, in these simple cases, the M coefficients as the percentage of pixels in one channel that intersect with some signal in other channel. The represented values are means + SEM (n = 8 fields; *P<0.05; **P<0.01; ***P<0.001). Scale bars represent 20 µm. (D) Analysis of molecular markers for autophagy and lysosomes. Western blot analysis of wild-type (WT), TIA1 KO and TIAR KO MEFs cell extracts using 2 µl from 1×10^7^ cells/ml to quantify the expression levels of LC3B and LAMP1 proteins, respectively. The blot was probed with antibodies against LC3B, LAMP1 and α-tubulin proteins, as indicated. Molecular weight markers and the identities of protein bands are shown. (E) Autophagic flux analysis. Obvious double-membraned autophagosome and vacuoles with engulfed bulk cytoplasm and cytoplasmic organelles (mitophagy) are shown. Please, see also Fig. S7 and S8. Scale bars represent 250 nm.

As shown above, TIA1 and TIAR-knocked-down MEF cells were much larger in size and had a flattened shape (Fig. 4C), a feature of cells in senescence. To determine whether TIA1 and TIAR silencing causes cell senescence, we examined the expression of senescence-associated β-galactosidase (SA-β-GAL), a classic biochemical marker for cellular senescence. We found that only 0.1% and 0.2% of TIA1 and TIAR knocked out MEF, respectively, were positively stained (Fig. 7A), ruling out a significant role of senescence in the altered size, shape and cell cycle delay observed in TIA1 and TIAR deficient cells.

### Increased cell death in TIA1 and TIAR knocked MEF cells

To investigate the mechanism underlying the cell proliferation and growth defects observed in TIA1 and TIAR deficient MEFs, as well as whether there is any effect on cell viability as a result of the rise of mitochondrial ROS production and DNA damage seen in these cells compared to WT MEFs, we assessed the rates of cell death of the TIA1- and TIAR-knocked out MEFs by 7-amino-actinomycin (7-AAD) and PE-Annexin V staining followed by FACS analysis. The results show that there is a little increase in the percentage of cell undergoing cell death in TIA1 and TIAR MEFs compared to WT MEF cells (Fig. 7B). Taken together, these observations suggest the existence of a putative regulatory mechanism to counteract cell death and promote survival response. Given that H_2_O_2_ is the most common and stable form of ROS, we decided to test its effects at short- and long-term on WT, TIA1 KO and TIAR KO MEFs by analyzing cell death and/or survival rates by flow cytometry. The observations indicated that the rates of cell survival among WT, TIA1 KO and TIAR KO MEFs were similar under lower doses (0.1–1 mM) of hydrogen peroxide, whereas the high rates of cell death in TIA1-knocked out MEFs versus WT MEFs were slightly lower with highest doses (10–20 mM) of H_2_O_2_ used for 6 hours (Fig. S9A). At long-term, the treatments of WT, TIA1 KO and TIAR KO MEFs with low doses (0.1–1.0 mM H_2_O_2_) for 3 days had inhibitory effects on cell proliferation (Fig. S9B), the incubation with 1 mM H_2_O_2_ suggested again the existence of a protective/adaptive survival mechanism associated prevalently to MEF cells lacking TIA1 or TIAR proteins (Fig. S9C).

### TIA1 and TIAR knocked MEF cells shows high rates of autophagy

Autophagy is an adaptive pro-survival program under cellular stresses [39], [40]. There is a growing evidence indicating that autophagy is activated in cellular situation in which cell cycle is delayed [39]. Therefore, we next investigated whether the deficiency of TIA proteins triggers autophagy. During autophagy, microtubule-associated proteins light chain 3-I (LC3-I) is converted to phosphatidylethanolamine-conjugated LC3-II, which associates with autophagic vesicles. The fusion of autophagosomes with lysosomes, classical hallmarks of autophagy, can be measured by assessing the colocalization of LC3B and LAMP1 (a lysosomal marker) [39]. As shown in Figure 7C and D, LC3B-II expression in TIA1 and TIAR KO MEFs was up-regulated compared to that in WT MEFs. This increase in LC3B-II expression correlated with the formation of autophagosomes, since colocalization between autophagosomes (positive to LC3B labeling) and lysosomes (positive to LAMP1 labeling) was increased in TIA1 and TIAR-knocked MEF in agreement with Manders' M1 and M2 colocalization coefficients, thus indicating the fusion between autophagosomes and lysosomes to generate autolysosomes (Fig. 7C and D). By transmission electron microscopy, we further observed a representative enrichment of double-membrane autophagosomes and vacuoles with engulfed bulk cytoplasm and cytoplasmic organelles in MEF lacking TIA1 and TIAR versus WT MEF (Fig. 7E and Fig. S7 and S8).

**Figure 8:**
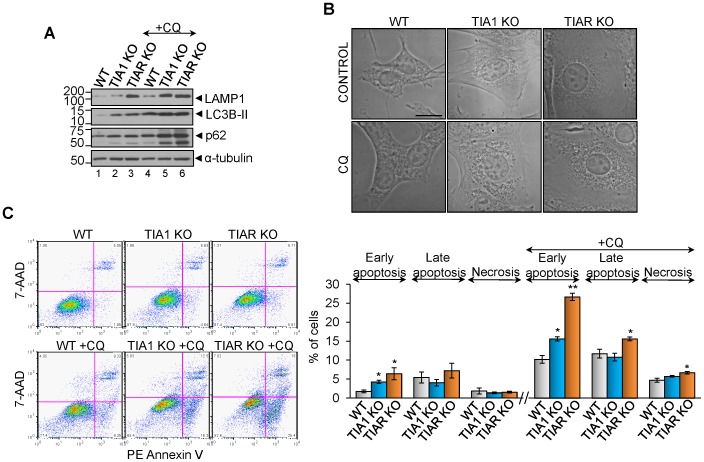
Autophagy inhibition by chloroquine increases the apoptotic rates in TIA1 and TIAR KO MEFs. (A) Analysis by Western blot of LAMP1, LC3B, p62 and α-tubulin protein expression upon chloroquine treatment of WT, TIA1 KO and TIAR KO MEFs by using 2 µl from 1×10^7^ cells/ml. (B) Representative micrographs by phase contrast of above chloroquine (CQ)-treated MEFs. Scale bar 20 µm. (C) Rates of cell death of chloroquine-treated WT, TIA1 KO and TIAR KO MEFs by flow citometry analysis. The represented values are means + SEM (n = 3; *P<0.05; **P<0.001).

To test the contribution of autophagy to the cell survival responses found in TIA1/TIAR-depleted MEFs versus WT MEFs, these cells were treated with 10 µM chloroquine (CQ), a drug that blocks the autophagic flux by inhibiting the fusion between autophagosomes with lysosomes. Above CQ-treated cells for 96 h were analyzed by Western blotting with anti-LC3B and anti-p62/SQSTM1 antibodies [41]. As expected, a rise of LC3B-II and p62 amounts was observed in the presence of the inhibitor (Fig. 8A), according to the effects promoted by chloroquine on autophagy. Interestingly, p62 protein didn't decrease in TIA1 and TIAR KO MEFs respect to WT MEFs (Fig. 8A), in agreement with oxidative-stress found in these cells [42]. By using confocal microscopy, cytoplasmic dense bodies were observed upon treatment with chloroquine (Fig. 8B). Additionally, we evaluated the rates of cell death associated to CQ-treated MEFs by staining of with 7-AAD and PE-Annexin V and posterior flow cytometry analysis. Early apoptosis (negative 7-AAD and positive PE-Annexin V) was enhanced in TIA1 and TIAR KO MEFs respect to WT MEFs, and late apoptosis (positive 7-AAD and positive PE-Annexin V) and necrosis (positive 7-AAD and negative PE-Annexin V) were enhanced in TIAR KO MEFs respect to WT MEFs (Fig. 8C). Taken together, these observations suggest that autophagy contributes to cell survival previously found in TIA1 and TIAR KO MEFs.

## Discussion

In this study, we have characterized the gene expression patterns and cellular phenotypes associated to the TIA1 and TIAR knocked-out murine embryonic fibroblast cell lines. Our results revealed an alteration of expression programs governing development in agreement with high rates of lethality observed in TIA1 or TIAR knocked-out mice [18], [25]. Comparison of the gene expression profiles of TIA-deficient MEFs and WT MEFs allowed the identification of RNAs whose expression was affected in both TIA1 and TIAR KO MEFs and RNAs that were differentially expressed in either TIA-deficient MEFs. Many of these non-coding and protein-coding RNAs are involved in tissue morphogenesis and differentiation during embryogenesis, including bone and cartilage formation, and heart, lung, muscle, skin, gonads, eye, nervous system developing (Fig. 1–3 and Fig. S1–S5). These RNAs can function as transcription factors, imprinting regulators, membrane receptors and cellular signalling and transduction kinases. Further, many of these regulated genes involved in embryogenesis are also implicated in cancer progression and cell adhesion, migration and angiogenesis. These observations are consistent with alterations on the prototypical mesenchymal characteristics that are associated to embryonic fibroblasts at E11.5 or E13.5 developmental stages [18], [25]. These results highlight a key role of TIA proteins in development.

However, the particular role of either TIA1 or TIAR in these processes is complex because the phenotype of the deficiency and its penetrance are strain dependent. For example, targeted knockout of TIA1 results in embryonic lethality, but the penetrance is less than 50% in both BALB/c and C57BL/6 founders [18]. Nonetheless, targeted disruption of TIAR leads to 100% embryonic lethality in the BALB/c background and 90% in the C57BL/6 background [25]. Mice lacking both in TIA1 and TIAR die before embryonic day 7, suggesting that one or both proteins must be expressed for normal embryonic development. These observations further support the independent role of either TIA1 or TIAR in controlling some key developmental processes. There are additional unique features associated to these proteins because TIA1-deficient mice are fully fertile, whereas TIAR-deficient mice that survive to birth are sterile due to defective germ-cell maturation [18], [25]. In this regard, we have identified a greater number of up- and down-regulated RNAs associated to TIAR KO MEFs cells than TIA1 KO MEFs as well as a set of down-regulated RNAs in TIAR KO MEFs associated to the gonadotrophin-releasing hormone (GnRH) signalling pathway (Fig. S5), which is involved in reproductive function and fertility [25]. Moreover, TIAR is solely required for normal germ-cell maturation [25]. Thus, these results suggest that these regulators are partially overlapping and redundant, but at the same time, can also show unique functional features. Interestingly, a recent study has provided new evidence on the role of TIAR during mouse embryogenesis using an animal gain-of-function model. This report showed that TIAR controls embryo late pre-implantation stages and that its overexpression significantly impaired embryonic development beyond implantation, thereby revealing the requirement of tightly controlled TIAR expression levels for normal mouse embryo development [26]. The altered gene expression patterns associated to processes such as cell adhesion, differentiation, angiogenesis, apoptosis, proliferation and intracellular signal transduction suggest that TIA1 and TIAR play, individually and collectively, essential roles as master organizers of gene networks controlling embryonic outgrowth and patterning. In fact, TIA proteins are involved in anterior/posterior and dorsal/ventral pattern formation (Fig. S1–S5). This idea might be consistent with a role for these proteins in early and late stages of embryo development and organ differentiation.

In molecular terms, the changes in expression levels of modulated RNAs caused by TIA1 and TIAR-deficiency might probably be mediated not by a unique mechanism, but rather by a combination of transcriptional and post-transcriptional mechanisms, including transcriptional rates, alternative and constitutive splicing events, RNA stability/turnover and/or translational efficiency of non-coding and protein-coding RNAs, given the pleiotropic effects of TIA proteins in RNA biology [6]–[22]. So far, only RNA maps associated to the TIA1 and TIAR proteins have been established in HeLa cells [13]. However, it is interesting to note that between human and mice only 10–20% of post-transcriptional regulatory events are conserved [13]. Therefore, TIA1 and TIAR knockout MEFs could be an interesting model to get new insights into how transcriptional and post-transcriptional activities of TIA proteins interplay to contribute to the regulation of gene expression during development and evolution [3]–[5].

Our results have shown that either TIA1 or TIAR deficiency in MEFs cause a significant reduction in cell proliferation, a delay in cell-cycle progression, defective mitosis, and abnormal cell size and shape. Moreover, these cells have altered mitochondria physiology, including increase number of mitochondria, altered morphology and increased membrane potential. These cells also produce excess cellular ROS and have massive DNA oxidative damage although it seems not seems to significantly affect cell viability (Fig. 4–7). Indeed, despite these cellular phenotypes apparently so deleterious to the cell associated to the TIA1- and TIAR-deficiency in MEFs, we have detected very modest rates of apoptosis and necrosis but instead we have observed high rates of autophagy, suggesting that autophagic responses might be able to shape survival mechanisms. In fact, genome-wide expression patterns show differentially expressed gene categories associated to phagosomes and lysosomes both in TIA1 and TIAR knockout MEFs (Fig. 1–3). Autophagy is a process by which normal and stressed cells engulf and sequester into autophagosome some cytosolic materials such as intracellular proteins and organelles that are subsequently degraded. This phenomenon occurs continuously under normal conditions to remove and recycle damaged proteins and organelles as a method of quality control [39]–[44]. Thus, mitophagy is a mechanism to limit ROS levels [45]. Hence, mROS and mitophagy can form a feedback loop, whereby mROS induce mitophagy, which limits further production of ROS by reducing mitochondria quantity. However, recent evidences indicate that autophagy response could also have a cell-killing but not cell-protective role under some cellular stresses, which leads to autophagic cell death. This method of controlled cell death is different to apoptosis [39], [40], [44], [45]. Several reports have pointed out the critical role of mROS production and together with the effects of cumulative oxidative damage and nuclear mitochondrial mutations leading to abnormalities of organismal and cellular function, altering cell metabolism and decreasing cell proliferation in yeast, mice and humans [45]. Therefore, it is tempting to speculate that this cellular scenario would be operating in MEF lacking TIA1 and TIAR proteins involving quantity of mitochondria and quantity of damaged mitochondria that produce more ROS. Thus, mitochondrial membrane potential appears to directly correlate with ROS production; therefore, anomalous mitochondrial population increases mitochondrial membrane potential, increase ROS levels, triggers DNA oxidation and damaged DNA-associated G2/M checkpoint promoting defective resolution of the cell cycle with abortive cytokinesis events followed by a survival response mediated by autophagy. The molecular and cellular details underlying TIA's effects on stress and/or survival potentially phenotypes should be elucidated in future studies.

## Conclusions

TIA1 and TIAR are two RNA binding proteins which have been involved in the control of gene expression in humans and mice. Mice lacking either TIA1 or TIAR proteins, as well as ectopically over-expressing TIAR, show higher rates of embryonic lethality [18], [25], [26]. The purpose of this study was to elucidate the effects of TIA1 and TIAR knockout on murine embryonic fibroblast (MEF) cells. The results illustrate that inactivation of TIA-proteins is sufficient to alter normal developmental program and signalling pathways in MEF cells. Our data indicate a broad alteration in cellular physiology caused by TIA1 or TIAR deficiency. Thus TIA1 and TIAR KO MEFs showed decreased rates of cell proliferation, alterations in cell cycle, increased cellular size and shape, abnormal mitochondrial populations, increased levels of mitochondrial membrane potential and ROS production and oxidative DNA damage, but low rates of cell death. Additionally, we have observed increased rates of autophagy in both TIA1 and TIAR KO MEFs, consistent with a severe cellular stress caused by either TIA1 or TIAR deficiency, suggesting that adaptive autophagy is induced as a survival mechanism.

## Supporting Information

Figure S1 **Summary of differentially expressed genes in TIA1 KO MEF.**
(XLS)

Figure S2 **Summary of differentially expressed genes in TIAR KO MEF.**
(XLS)

Figure S3 **List of shared and opposite expressed genes and their GO/KEGG analysis from TIA1 and TIAR KO MEFs.**
(XLS)

Figure S4 **Summary of GO and KEGG database analyses of microarray-predicted genes from TIA1 KO MEF.**
(XLS)

Figure S5 **Summary of GO and KEGG database analyses of microarray-predicted genes from TIAR KO MEF.**
(XLS)

Figure S6 **Validation of microarray-predicted changes by quantitative PCR and list of primer pair sequences used.**
(PDF)

Figure S7 **Summary of cellular microphotographs from WT, TIA1 KO and TIAR KO MEFs by electron microscopy.**
(PDF)

Figure S8 **Summary of microphotographs of mitochondrial populations from WT, TIA1 KO and TIAR KO MEFs by electron microscopy.**
(PDF)

Figure S9 **Effect of hydrogen peroxide (H_2_O_2_) treatment on cell death and/or survival in WT, TIA1 KO and TIAR KO MEFs.** (A) Rates of cell death (early and late apoptosis and necrosis) and survival after 6 hr treatment with none, 0.1, 1, 10, and 20 mM H_2_O_2_ were quantified by using 7-AAD staining and PE Annexin V apoptosis detection kit followed by flow cytometry analysis. (B) Rates of cell proliferation after 3 d treatment with none, 0.1, 1, and 10 mM H_2_O_2_ by direct count of cell number. (C) Rates of cell death (early and late apoptosis and necrosis) and survival after 3 days treatment with none, 0.1, 1, and 10 mM H_2_O_2_ were quantified by using 7-AAD taining and PE Annexin V apoptosis detection kit followed by flow cytometry analysis. In all cases, error bars indicate the standard error of the mean (SEM; n = 2; *P<0.05; **P<0.01).
(PDF)
