# Supplementary material for: T-cell Intracellular Antigen (TIA)-Proteins Deficiency in Murine Embryonic Fibroblasts Alters Cell Cycle Progression and Induces Autophagy
Source: PLoS One. 2013 Sep 24;8(9):e75127. doi: 10.1371/journal.pone.0075127 (PMC3782481; doi:10.1371/journal.pone.0075127)
Supplement: Figure S6 — Validation of microarray-predicted changes by quantitative PCR and list of primer pair sequences used. (PDF) [file pone.0075127.s006.pdf]

QPCR - Primers

Gene	Forward	Reverse	Product length (bp)
ARG2	TGATGCATTTGACCCTAAACTG	ATCCAGAGCTGACAGCAACC	124
EREG	TGACAGTGATTCTCATTTTCCTG	TCTCATATTCCTCCCTCGATTT	111
FBN2	TCAATGGCTACACCAAGAAAGAT	TGGGCTGTCCATGGCTAC	110
GAPDH	CTCCCACTCTTCCACCTTCG	CATACCAGGAAATGAGCTTGACAA	77
HMGA2	AAGGCAGCAAAAACAAGAGC	CCGTTTTTCTCCAATGGTCT	68
MEST	CTCTGTCTGTCGAATGGAGGT	CGTCTTTGAGGAGCTTTTGG	73
SFRP1	ATGTGCTCCAGAAGCAGACC	GTCAGAGCAGCCAACATGC	60
SFRP2	AAACCCTTTGTAAAAATGACTTCG	CAGCTTGTAAATGGTCTTGCTC	113
TIA1	AGTTCCATGAGCACCGTCAT	TGTTGCCCAATTCACTTTCA	92
TIAR	GGAACCAACAAGGATTTGGA	GAGGAGGCTGAGCACCAA	75
XIST	CTACTGCTCCTCCGTTACATCA	AGGAGCACAAAACAGACTCCA	74

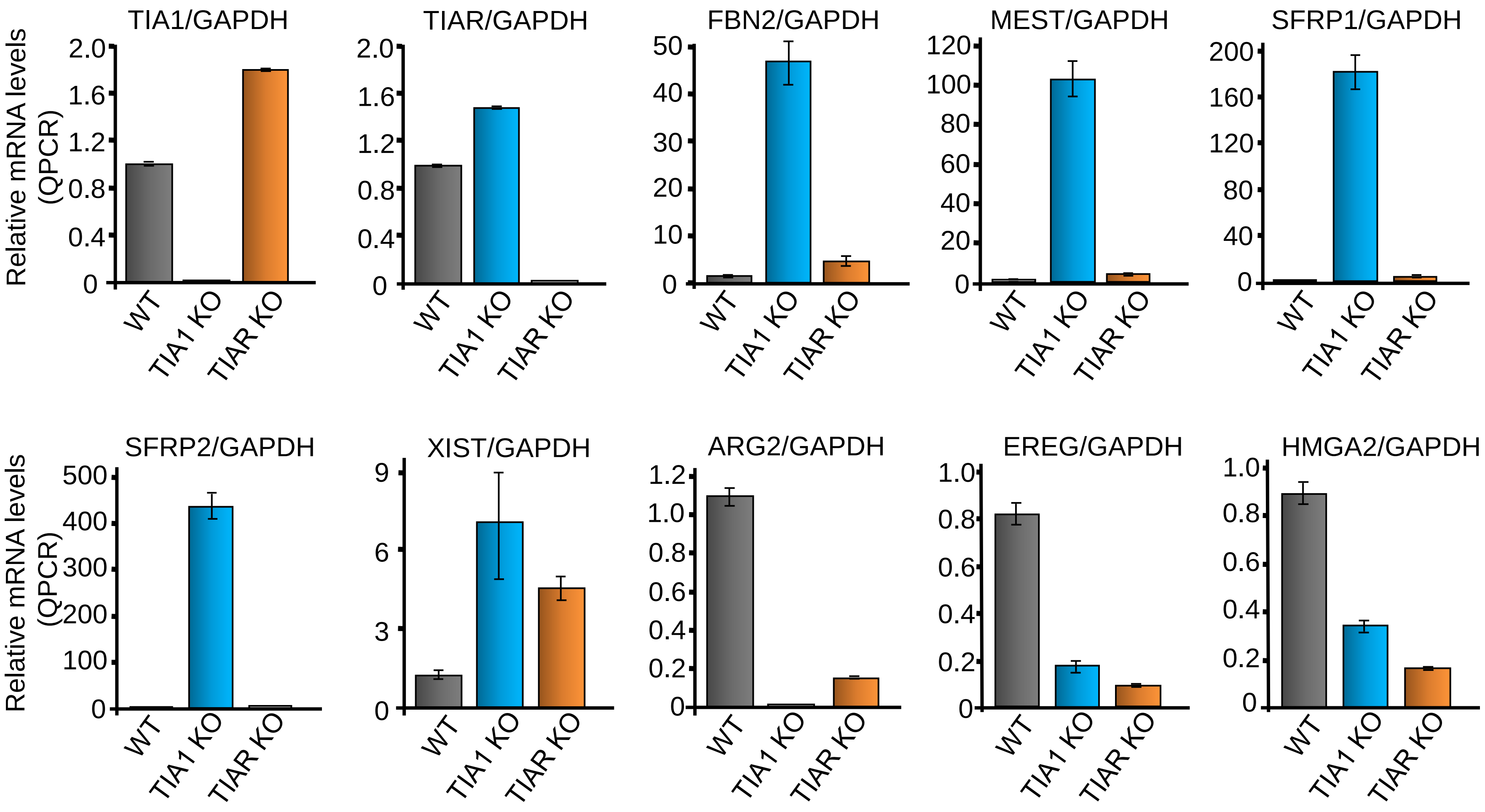
TIA1/GAPDH
TIAR/GAPDH
FBN2/GAPDH
MEST/GAPDH
SFRP1/GAPDH
SFRP2/GAPDH
XIST/GAPDH
ARG2/GAPDH
EREG/GAPDH
HMGA2/GAPDH
Relative mRNA levels (QPCR)
WT
TIA1 KO
TIAR KO
