# Supplementary material for: T-cell Intracellular Antigen (TIA)-Proteins Deficiency in Murine Embryonic Fibroblasts Alters Cell Cycle Progression and Induces Autophagy
Source: PLoS One. 2013 Sep 24;8(9):e75127. doi: 10.1371/journal.pone.0075127 (PMC3782481; doi:10.1371/journal.pone.0075127)
Supplement: Figure S8 — Summary of microphotographs of mitochondrial populations from WT, TIA1 KO and TIAR KO MEFs by electron microscopy. (PDF) [file pone.0075127.s008.pdf]

Electron micoscopy (12,000x)
WT MEF

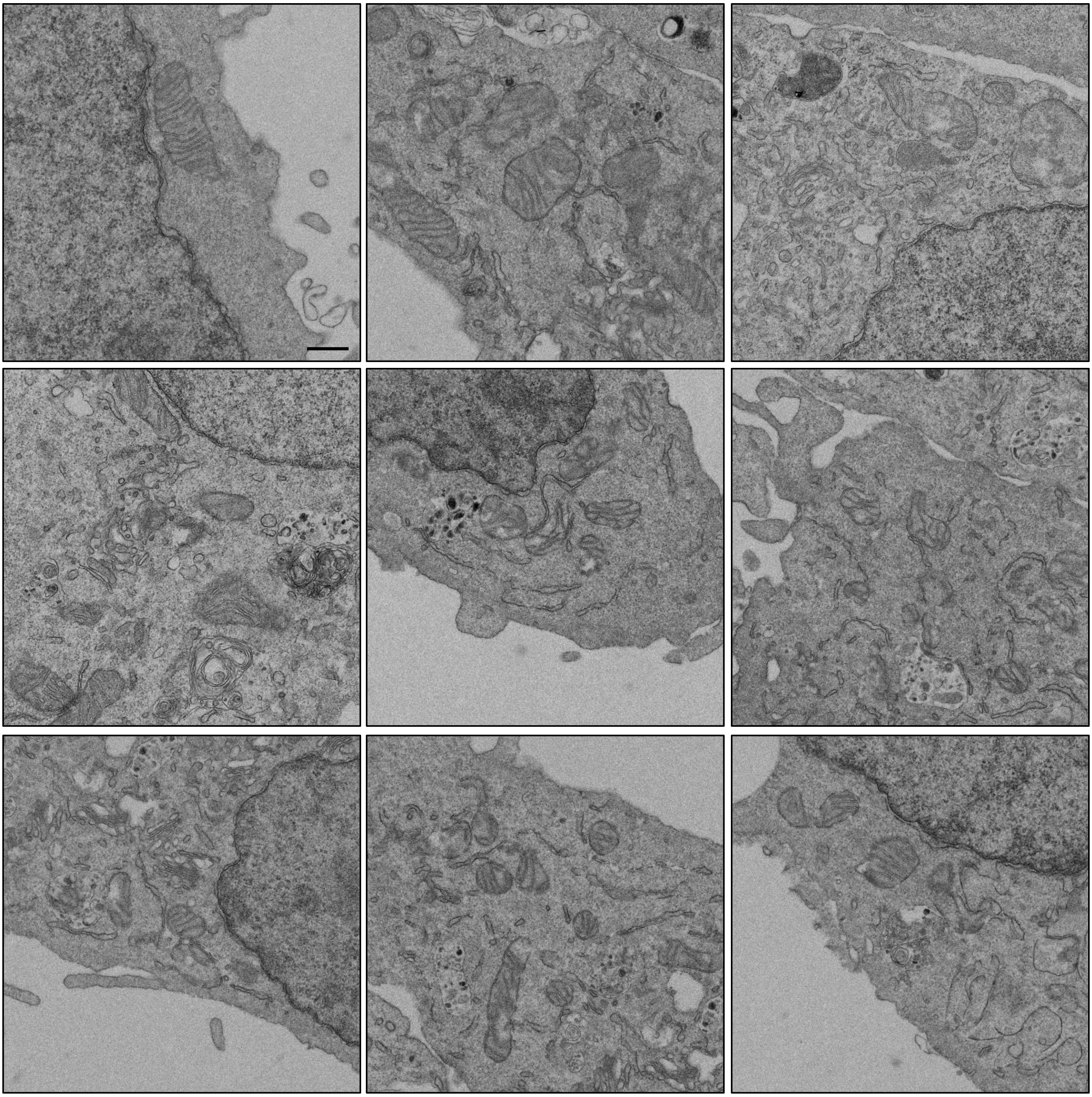

Scale bar = 500 nm

Electron micoscopy (12,000x)
TIA1 KO MEF

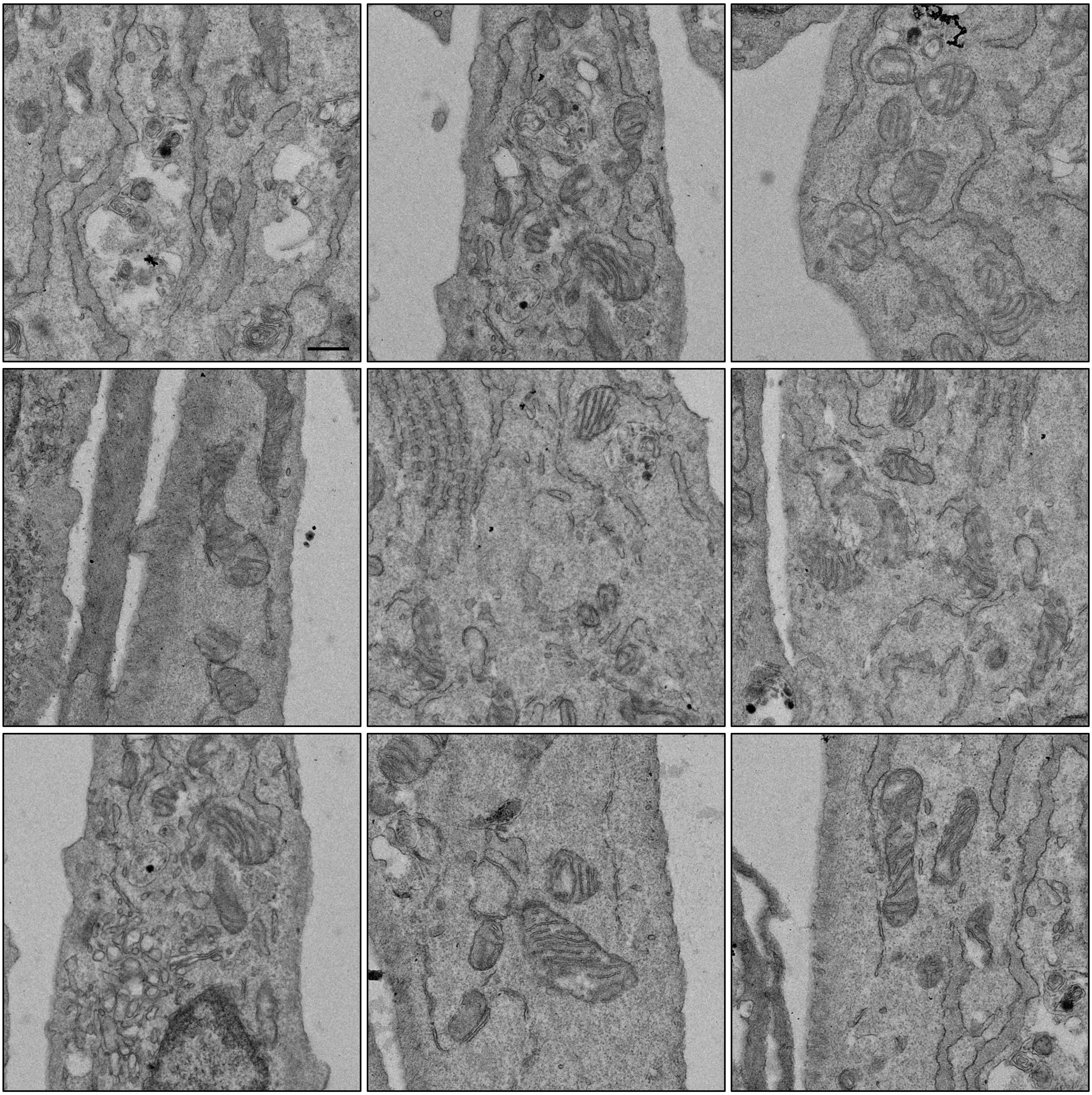

Scale bar = 500 nm

Electron micoscopy (12,000x)
TIAR KO MEF

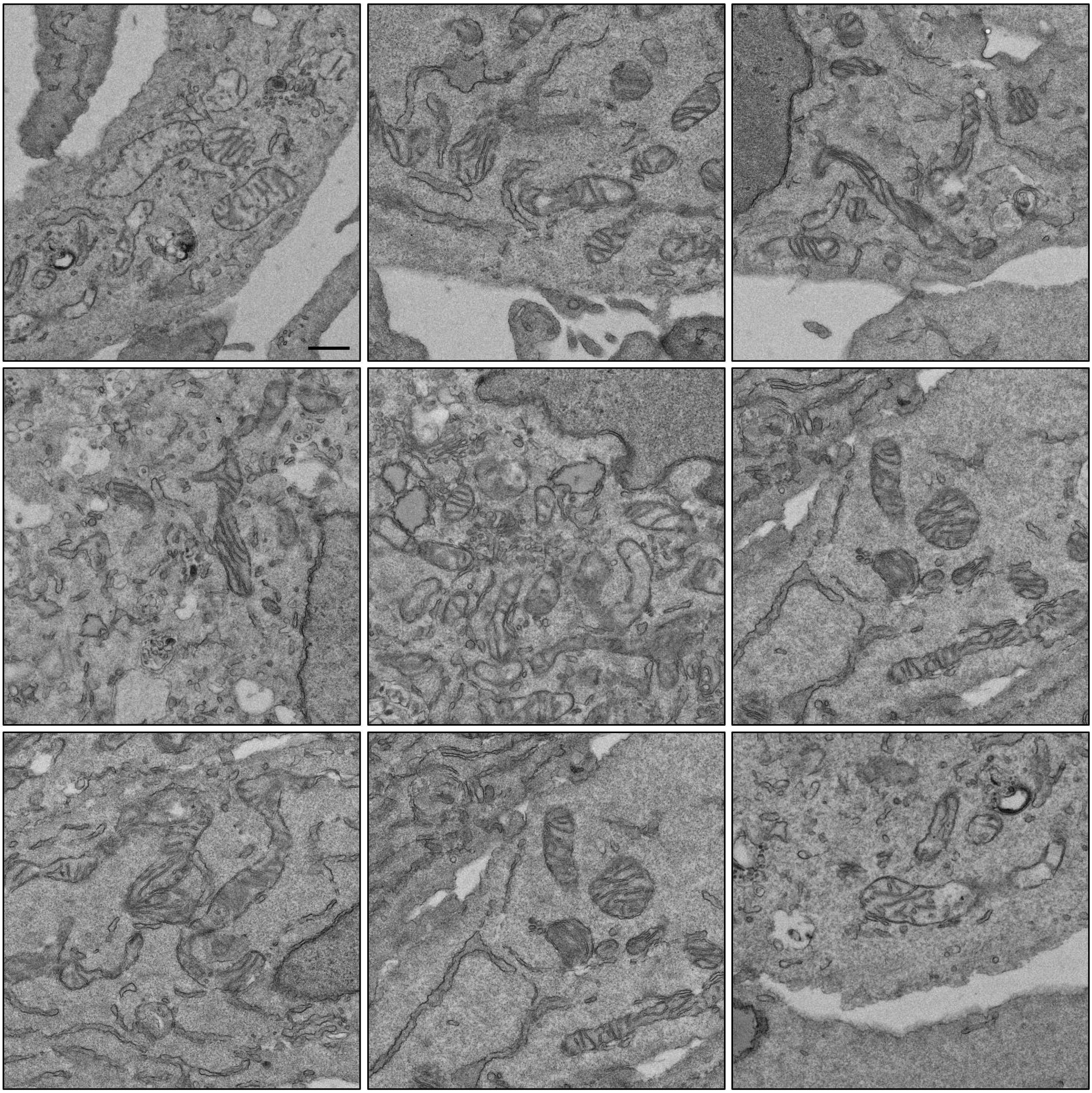

Scale bar = 500 nm
