# Supplementary figures and images for: T-cell Intracellular Antigen (TIA)-Proteins Deficiency in Murine Embryonic Fibroblasts Alters Cell Cycle Progression and Induces Autophagy
Source: PLoS One. 2013 Sep 24;8(9):e75127. doi: 10.1371/journal.pone.0075127 (PMC3782481; doi:10.1371/journal.pone.0075127)

**A**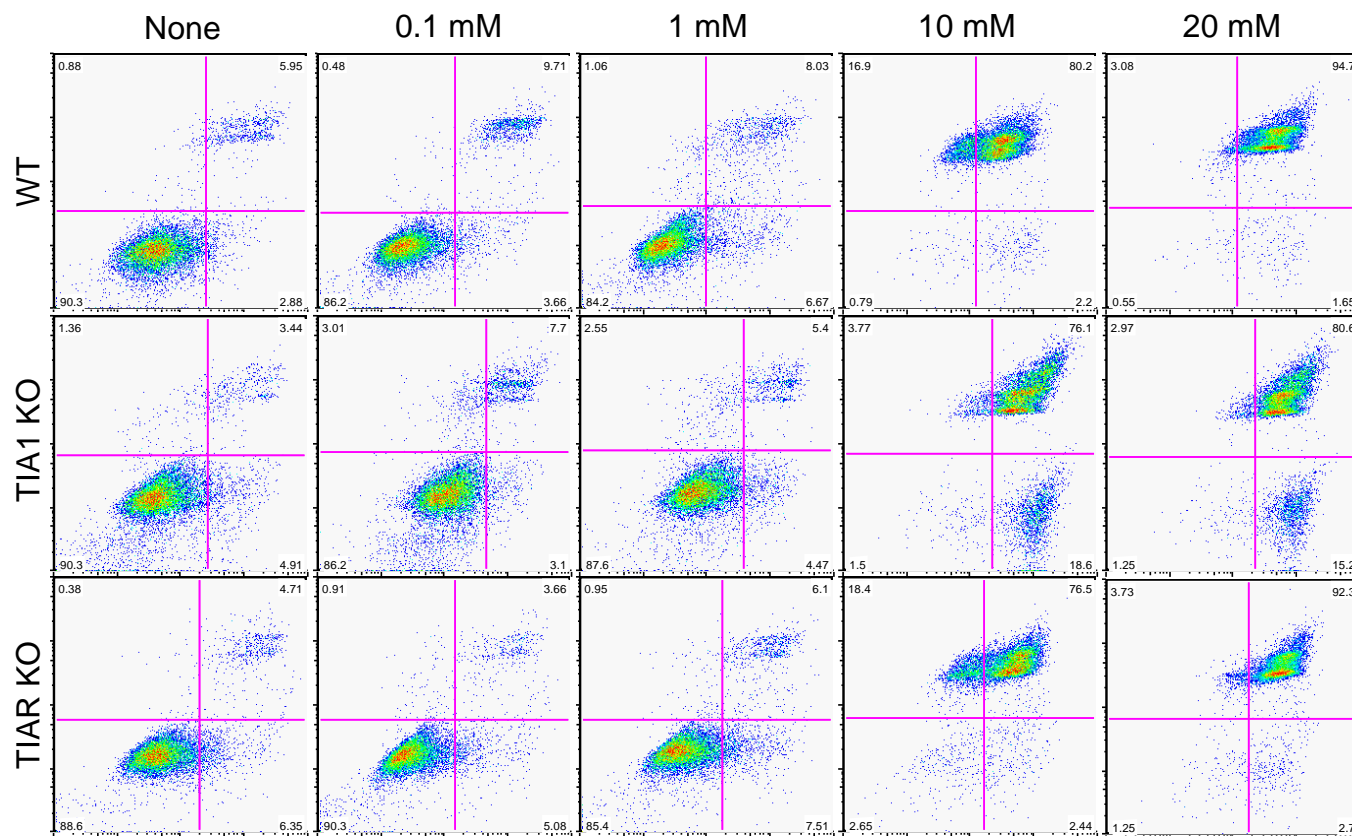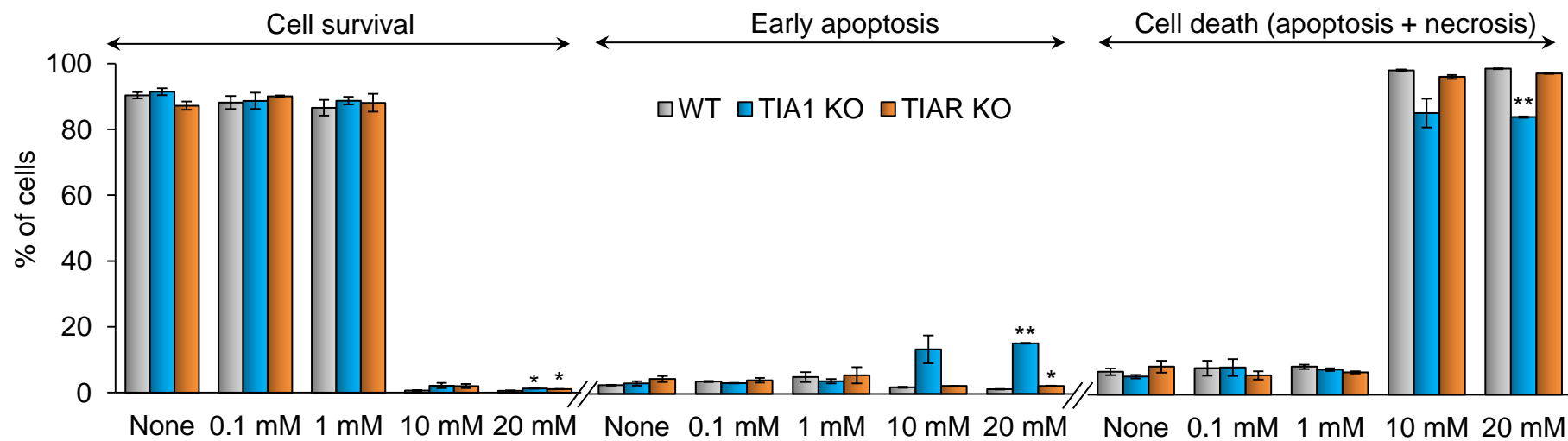

**B**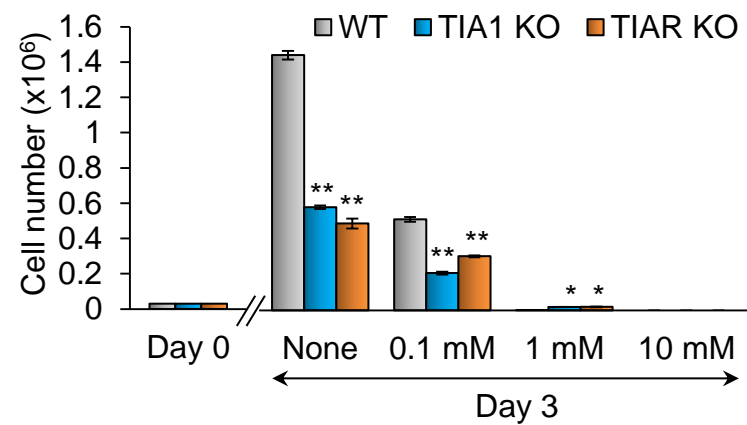**C**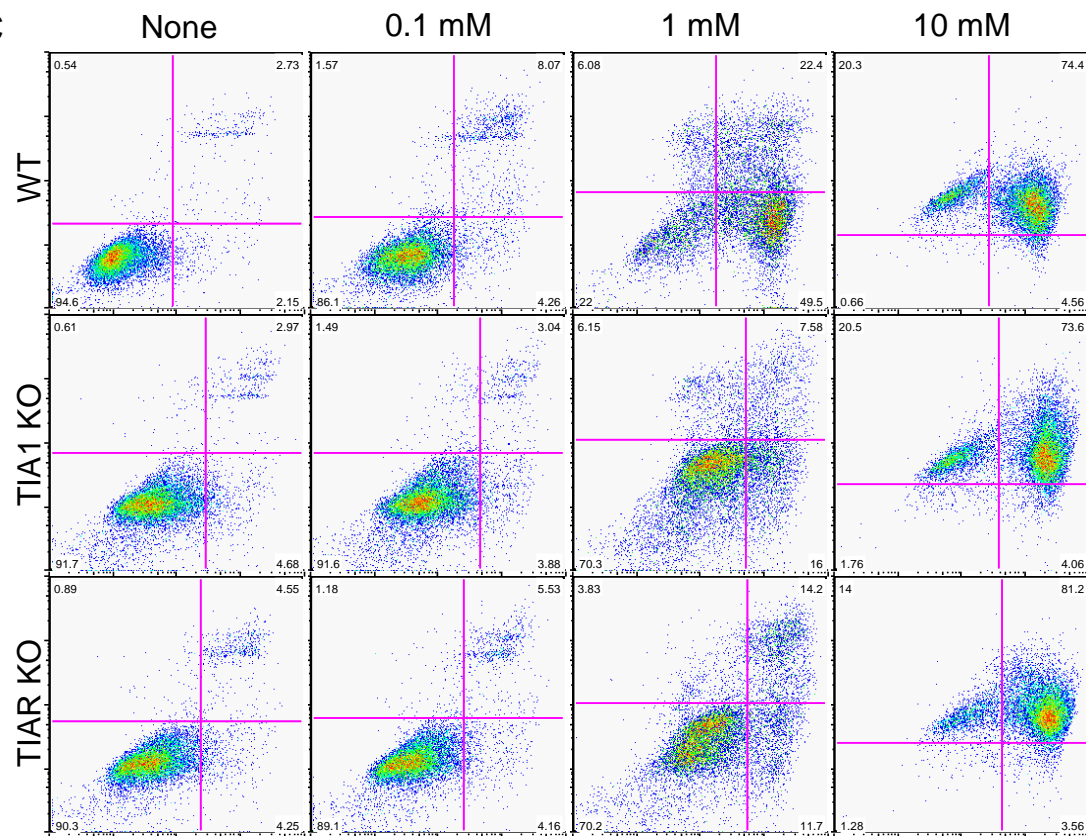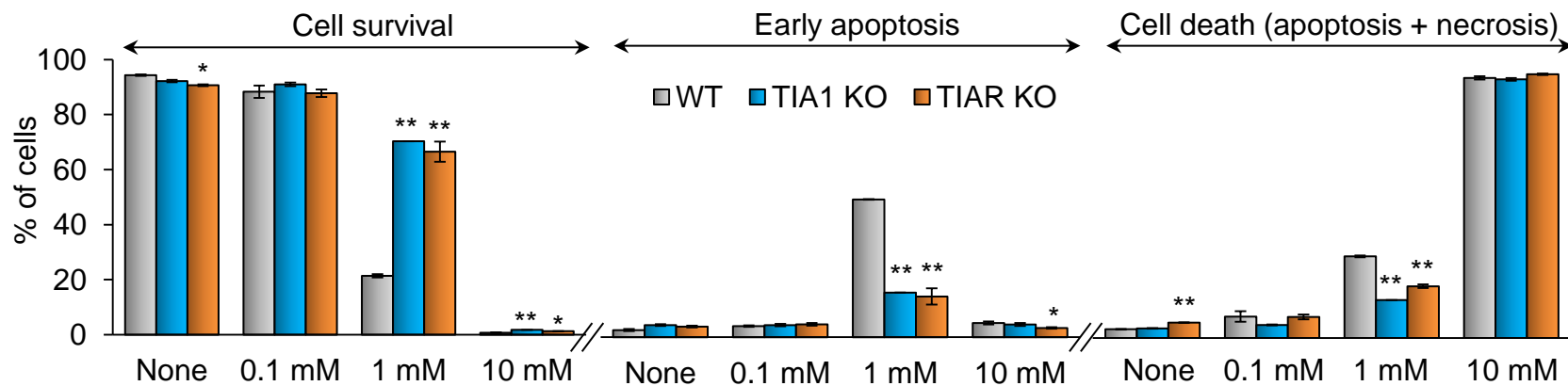

Supplement: Figure S9 — Effect of hydrogen peroxide (H2O2) treatment on cell death and/or survival in WT, TIA1 KO and TIAR KO MEFs. (A) Rates of cell death (early and late apoptosis and necrosis) and survival after 6 hr treatment with none, 0.1, 1, 10, and 20 mM H2O2 were quantified by using 7-AAD staining and PE Annexin V apoptosis detection kit followed by flow cytometry analysis. (B) Rates of cell proliferation after 3 d treatment with none, 0.1, 1, and 10 mM H2O2 by direct count of cell number. (C) Rates of cell death (early and late apoptosis and necrosis) and survival after 3 days treatment with none, 0.1, 1, and 10 mM H2O2 were quantified by using 7-AAD taining and PE Annexin V apoptosis detection kit followed by flow cytometry analysis. In all cases, error bars indicate the standard error of the mean (SEM; n = 2; *P<0.05; **P<0.01). (PDF) [file pone.0075127.s009.pdf]
