# Supplementary material for: T-cell Intracellular Antigen (TIA)-Proteins Deficiency in Murine Embryonic Fibroblasts Alters Cell Cycle Progression and Induces Autophagy
Source: PLoS One. 2013 Sep 24;8(9):e75127. doi: 10.1371/journal.pone.0075127 (PMC3782481; doi:10.1371/journal.pone.0075127)
Supplement: Figure S7 — Summary of cellular microphotographs from WT, TIA1 KO and TIAR KO MEFs by electron microscopy. (PDF) [file pone.0075127.s007.pdf]

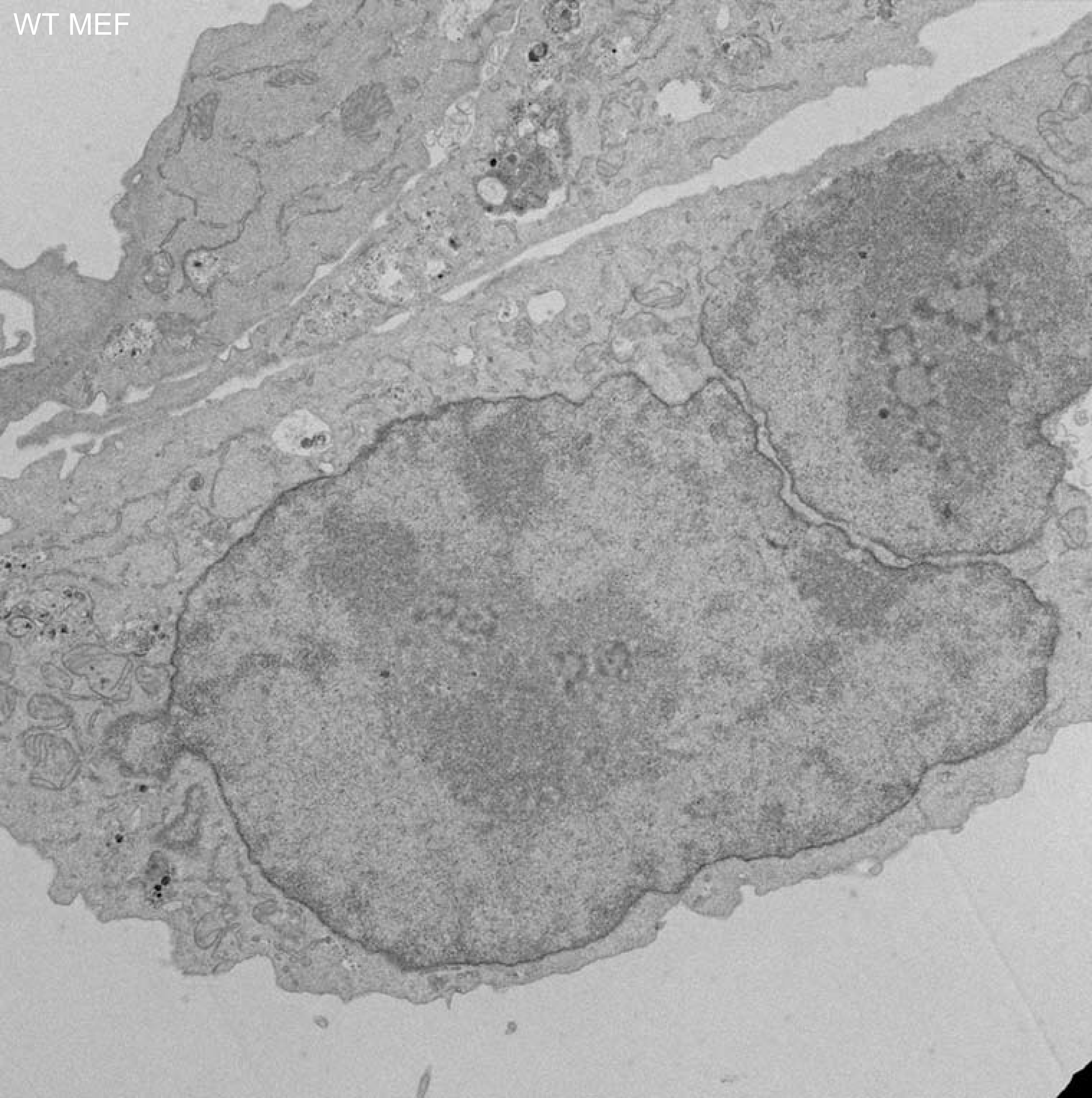
WT MEF

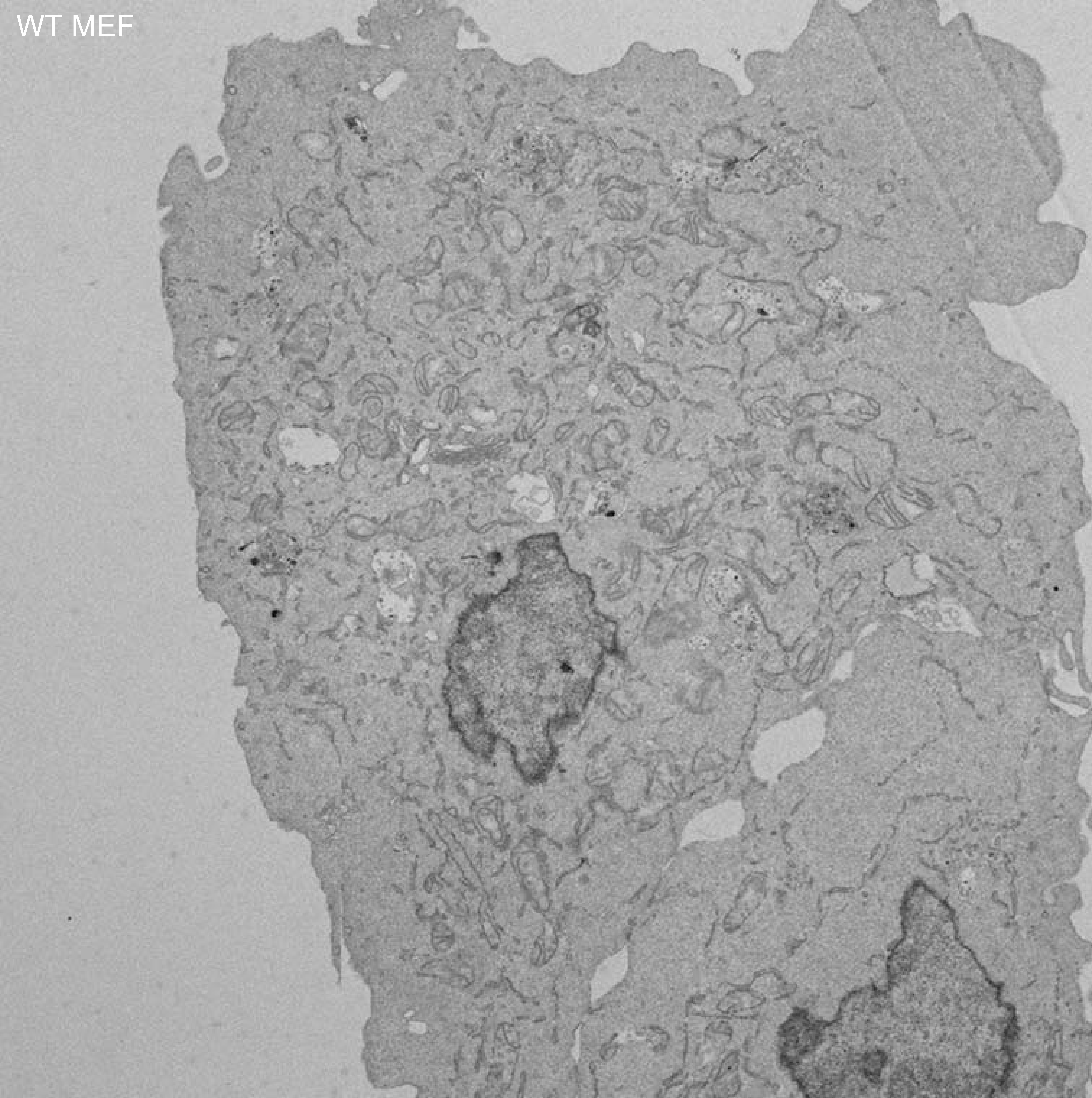
WT MEF

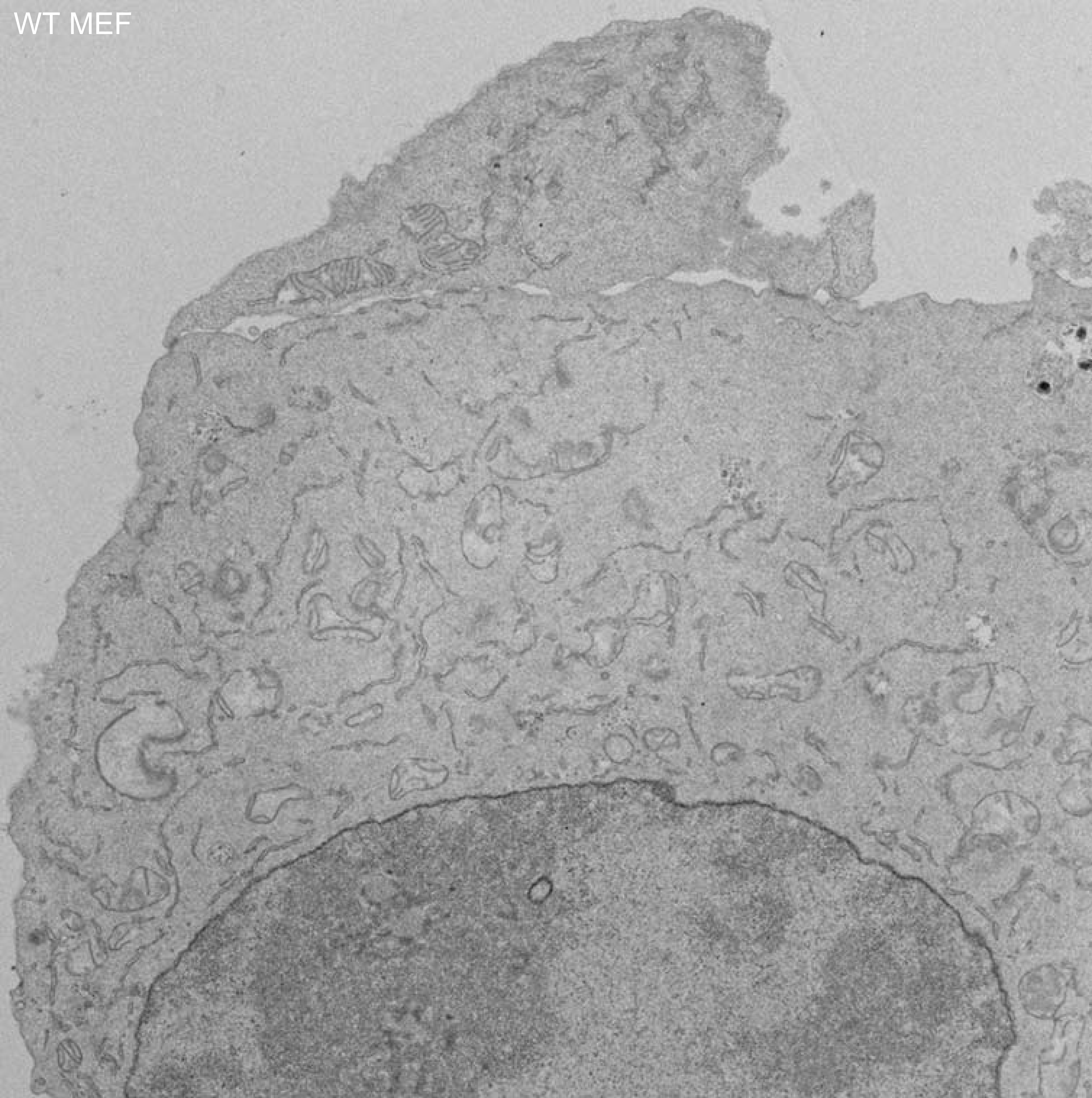
WT MEF

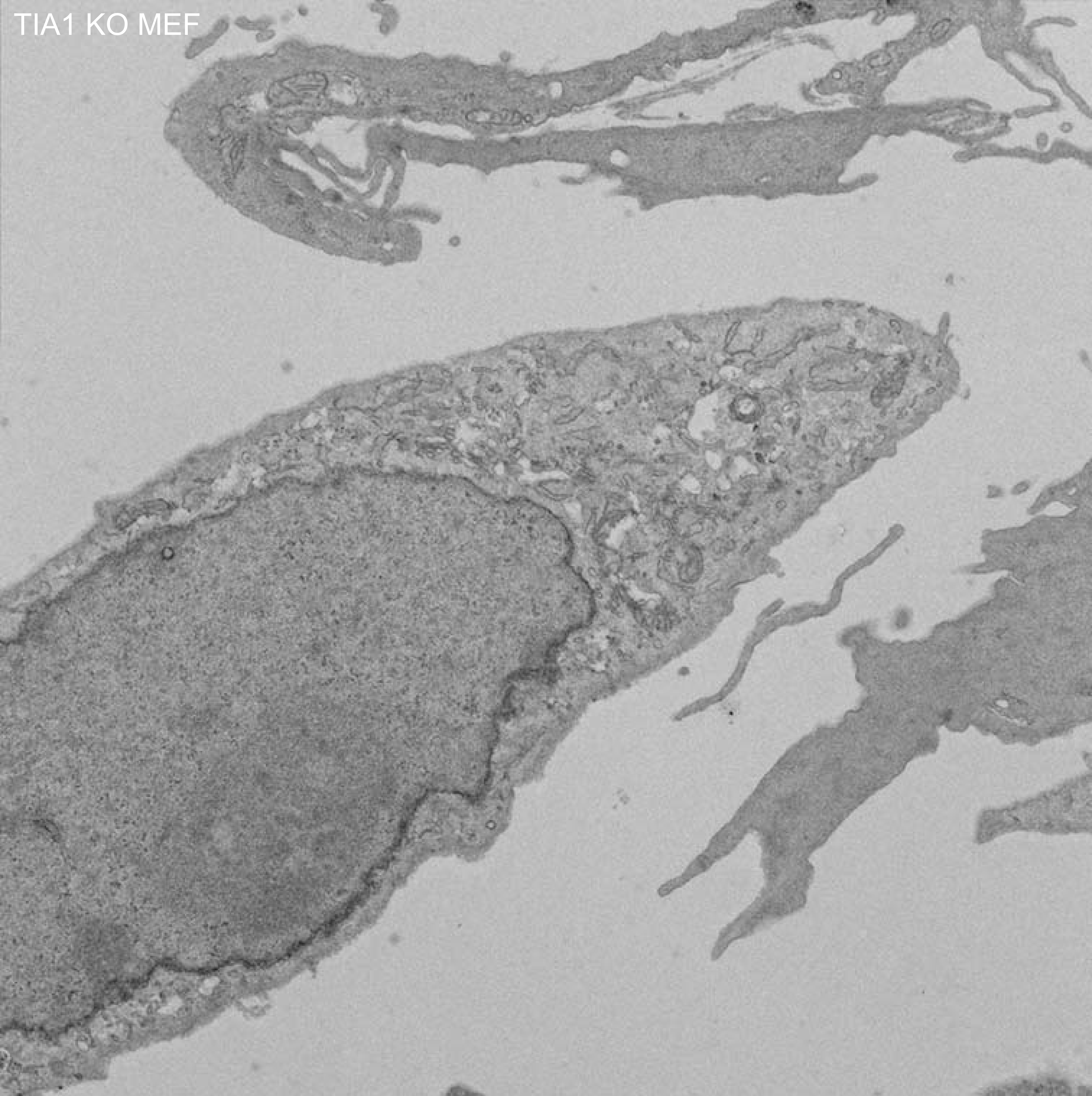
TIA1 KO MEF

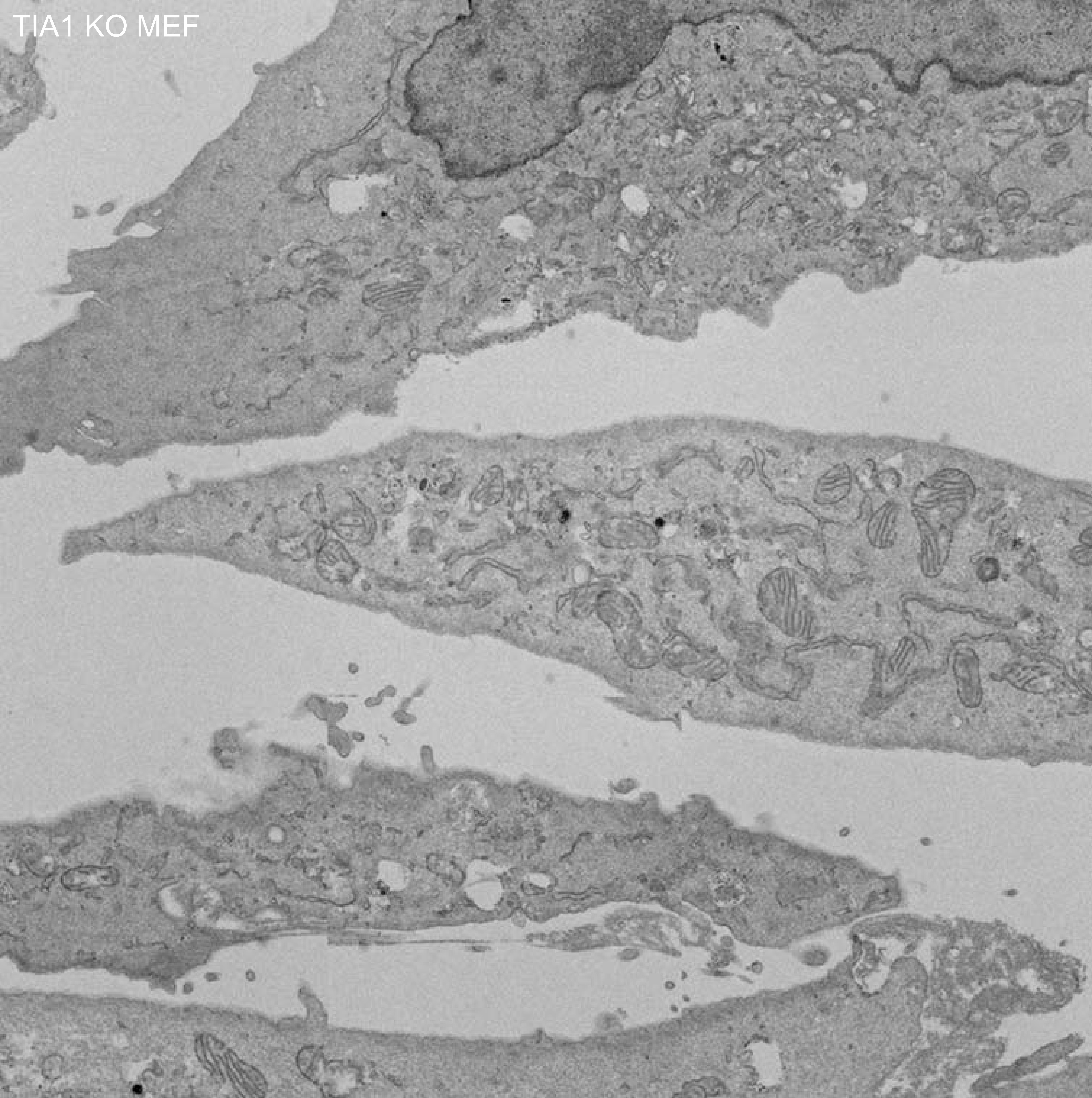
TIA1 KO MEF

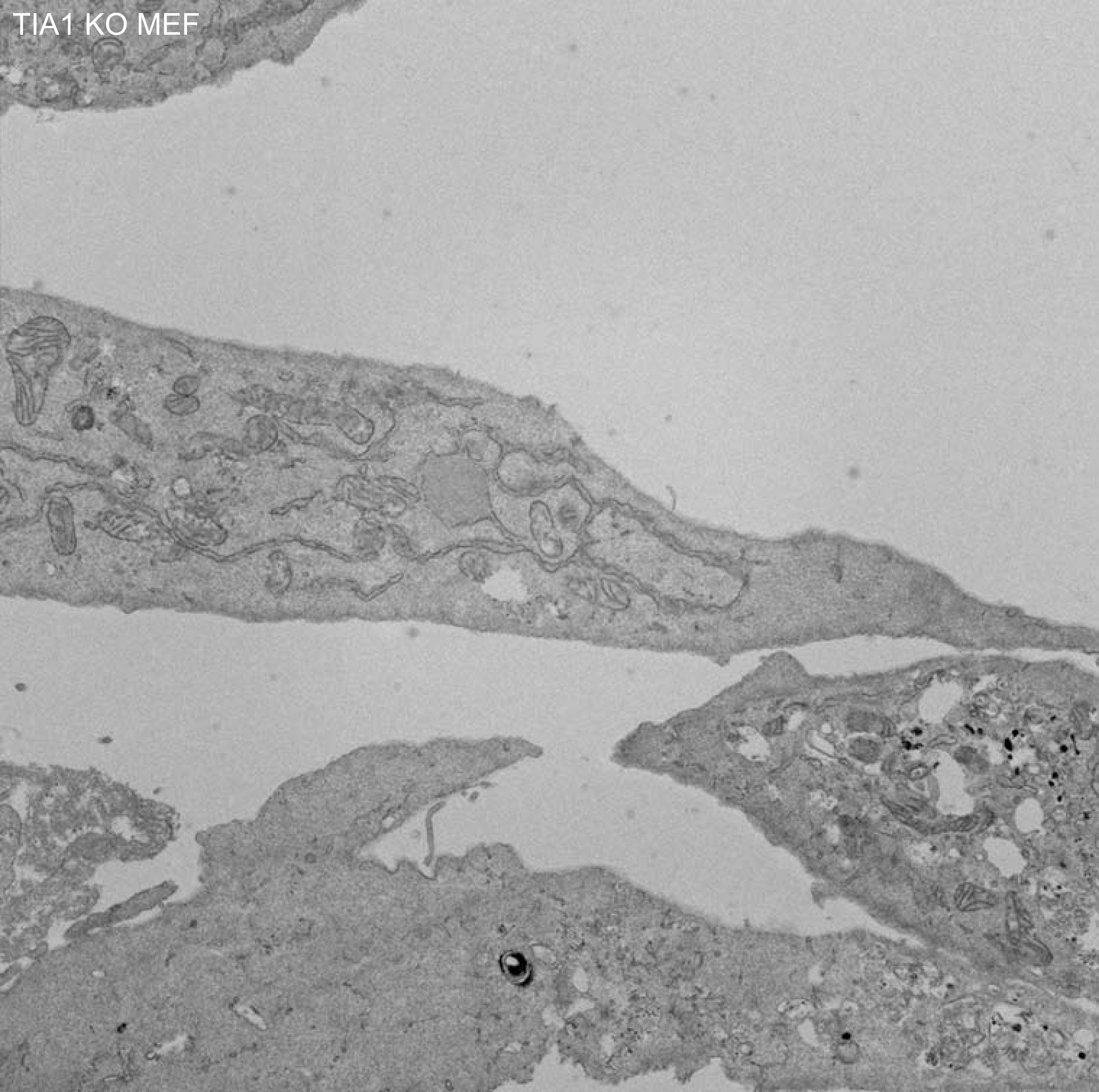
TIA1 KO MEF

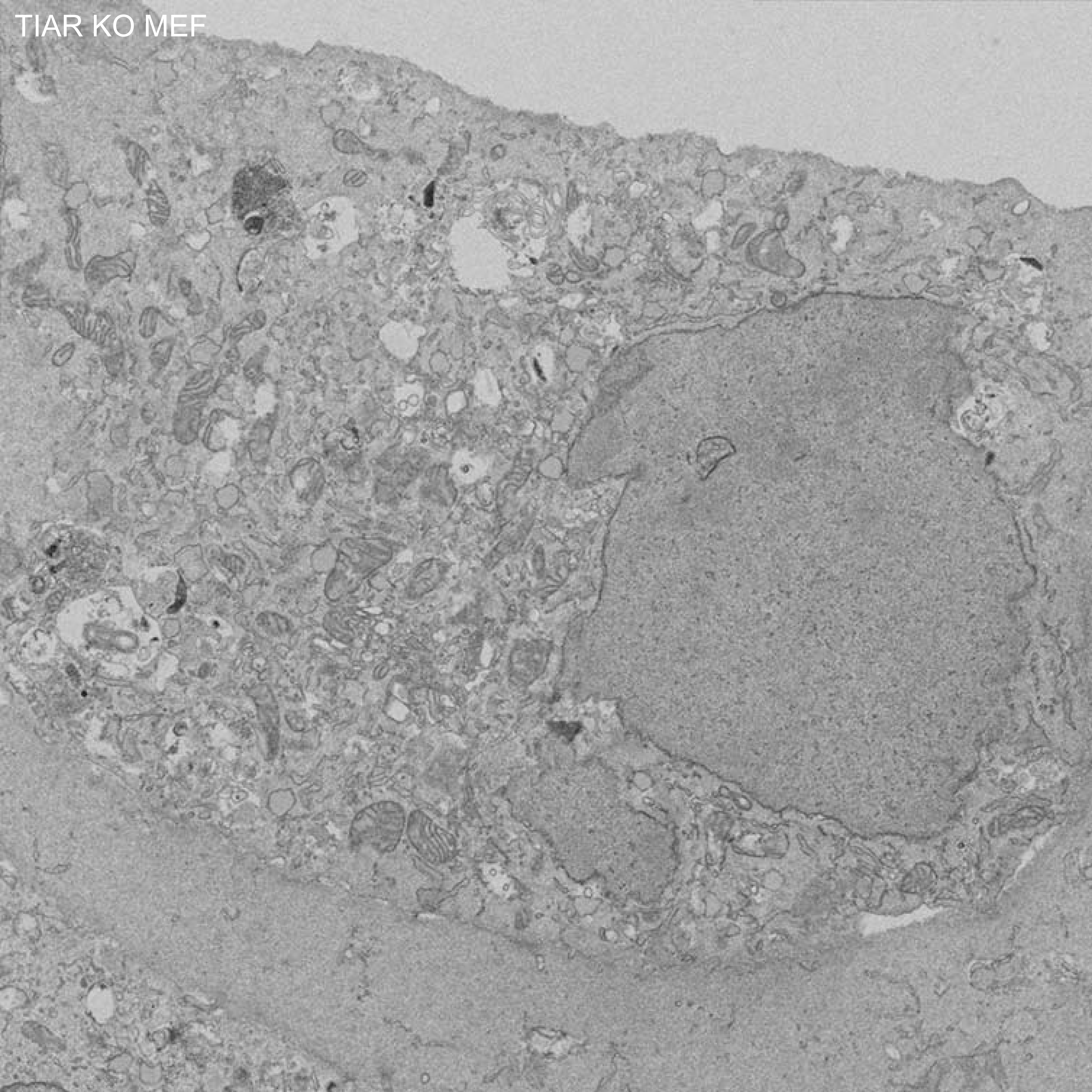
TIAR KO MEF

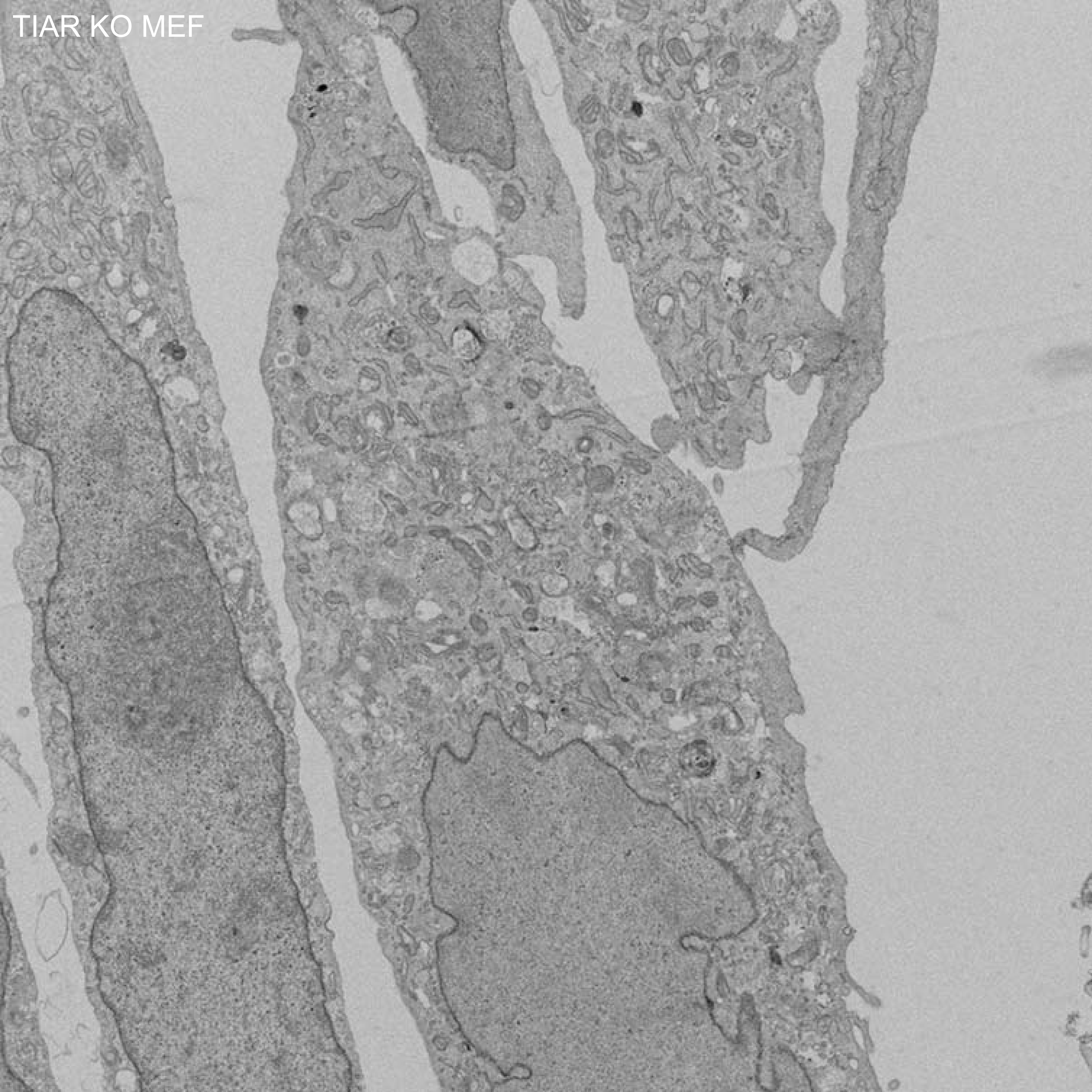
TIAR KO MEF

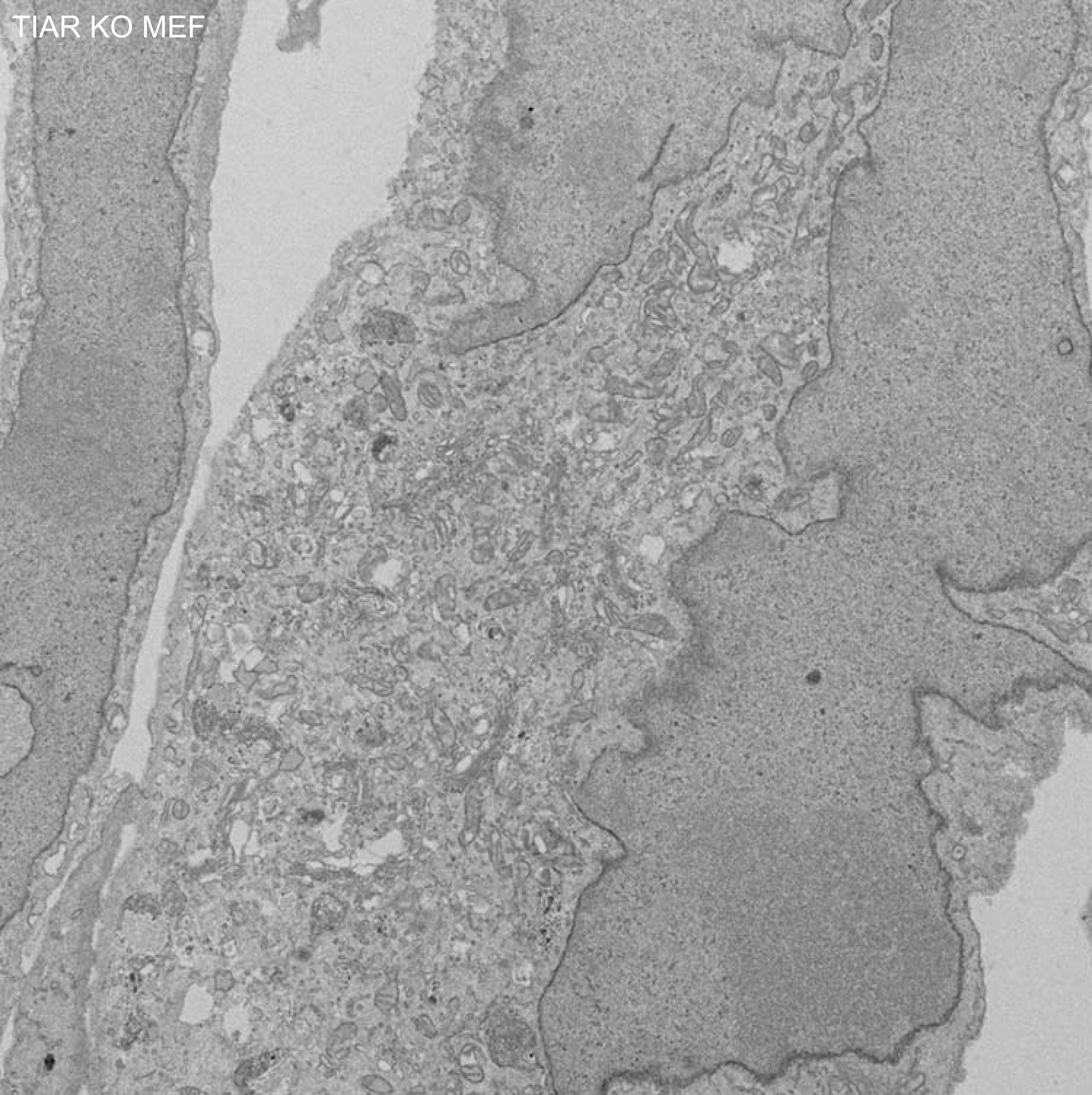
TIAR KO MEF
